# Intra articular hyaluronic acid in the management of knee osteoarthritis: Pharmaco-economic study from the perspective of the national health insurance system

**DOI:** 10.1371/journal.pone.0173683

**Published:** 2017-03-22

**Authors:** Thierry Thomas, Françoise Amouroux, Patrice Vincent

**Affiliations:** 1 Service Rhumatologie, CHU, St Etienne, France; 2 UFR Sciences Pharmaceutiques, Bordeaux, France; 3 LCA Pharmaceutical, Chartres, France; University of Umeå, SWEDEN

## Abstract

**Introduction:**

Pharmaco-economic data on the management of knee osteoarthritis (OA) with intra articular hyaluronic acid (IA HA) viscosupplementation is limited. We contrasted IA HA with non-steroidal anti-inflammatory drugs (NSAIDs).

**Methods:**

Observational, prospective and multicenter study comparing treatments of knee OA costs and efficacy with either NSAIDs alone, or hyaluronic acid (Arthrum H 2%^®^), during a 6-month follow-up period. The investigators were pharmacists who recorded data on disease, drug consumption and healthcare circuit. Retrospectively, the 6-month period preceding inclusion was also studied, to ensure the comparability of groups.

**Results:**

199 patients were analyzed in a NSAIDs group and 202 in an IA HA group. Any of the WOMAC sub-scores and the EQ-5D Quality of Life index were significantly improved in the IA HA group (p<0.0001) at 3 and 6 months. Clinical results were therefore in favor of the IA HA group. The total drug expenses per 6-month period were comparable before and after inclusion, €96 and €98 for NSAIDs group vs €94 and €101 for IA HA group, which indicates no evidence of additional cost from IA HA. For the active part of the population, the incidence of sick leave was lower in the IA HA group, indicating a better maintenance of patient activity. The overall expense on 12 months (6 months before and 6 months after inclusion) for the national health insurance system was comparable for NSAIDs and IA HA groups: €528 and €526, respectively. The number of patients taking NSAIDs significantly decreased in IA HA group (from 100% at inclusion to 66% at 1–3 months and 44% at 4–6 months), but remained unchanged (100%) during the follow-up period, in NSAIDs group.

**Conclusion:**

Treatment with IA HA did not generate additional cost for the national health insurance and was associated with a functional improvement of knee osteoarthritis and Quality of Life. The cost-utility analysis was in favor of IA HA, with a gain of QALY equivalent to half a month, after the 6-month follow-up period comparing both treatments. The NSAIDs consumption was decreased in the IA HA group, resulting in an improved estimated benefit/risk ratio.

## Introduction

Knee osteoarthritis (OA) is the first cause of chronic functional disabilities in developed countries with about 250 million people in the world suffering from knee OA [1]. In France, a recent population-based survey in people from 40 to 75 years reported that the prevalence of knee OA ranged from 2.1% (40–49 years) to 10.1% (70–75 years) for men and from 1.6 to 14.9% for women, respectively [2]. In the USA, the radiological prevalence of knee OA was estimated at 3.8% of the entire population [3]. From these results it could be estimated that there are 2–2.5 million people suffering from knee OA in France in 2010–2014. The impact of knee OA on quality of life by limiting activity is major; knee OA is one of the medical conditions accounting for the most severe physical disability in non-institutionalized elderly people [4].

Intra-articular administration of hyaluronic acid (IA HA) has been demonstrated to be as effective as NSAIDs and to induce fewer systemic adverse events [5–7]. IA HA improves viscoelasticity, lubrication and shock absorption properties of the synovial fluid. HA has a delayed onset of action in comparison with intra-articular corticosteroids, but a longer-lasting benefit [8]. It is widely recognized that viscosupplementation with IA HA, is a well-tolerated treatment [8–19], which is recommended [20,21]. Most of adverse effects reported are only local and limited to pain or swelling at injection point. These symptoms are generally resolved spontaneously within 1–2 days and ice application is necessary in some cases.

Knee OA has a major impact on healthcare costs [22,23]. However, only a few medicoeconomic studies have evaluated the cost of knee OA [24,27], with two of them from the perspective of the French national health insurance system [24,25].

The objectives of this study were a benefit–risk analysis (based on the assessment of NSAIDs consumption) and a cost-utility analysis (based on the assessment of treatment costs and health improvement) before and after administration of IA HA or alternatively continuing NSAIDs, under the conditions of every day practice.

## Methods

### Objectives of the study

The primary objective of the study was a benefit-risk analysis: to assess the consumption of NSAIDs and its evolution under IA HA. This was based on the assumption that NSAIDs are not free of iatrogenicity, and therefore any reduction in NSAIDs uptake (individually or globally) was considered as an improvement of the estimated benefit-risk ratio.

The secondary objective of the study was a cost-utility analysis: to assess the treatment costs in current practice and after intervention or not with IA HA; to assess health improvement and Quality of Life, associated with IA HA, in comparison with common practice with NSAIDs.

### Study design

**Fig 1:**
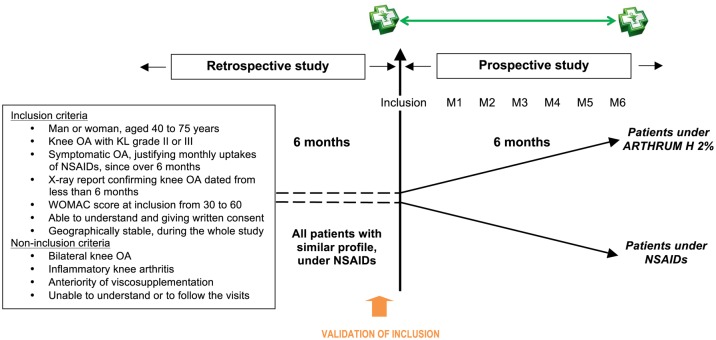
Study design. Patients with knee osteoarthritis were retrospectively assessed during 6 months at inclusion visit and prospectively during the next 6 months (one visit each month).

This was an observational (non-interventional), prospective and multicenter study (Fig 1) comparing the knee OA treatments, using NSAIDs alone, or IA HA viscosupplementation.

The IA HA viscosupplementation product used was Arthrum H 2%^®^, a highly concentrated solution containing 40mg HA per 2mL-syringe (Mw ≈ 2,500 kDa). The treatment consisted of 3 injections at one week intervals, always administrated by a specialist doctor [28,29]. One session treatment is typically proven to be efficient for one year [30].

**Fig 2:**
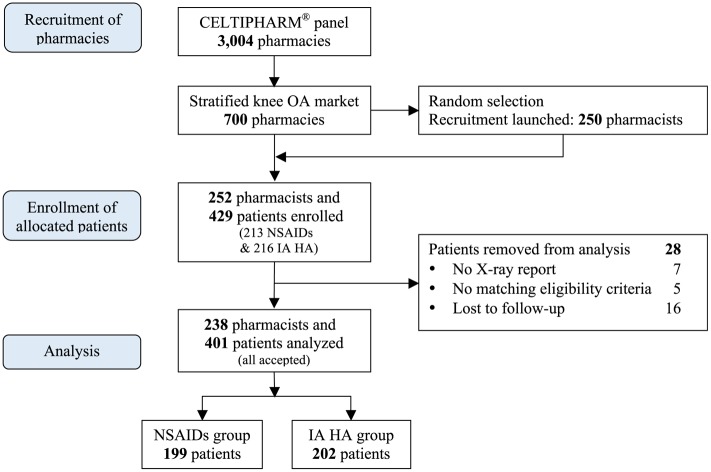
Study flow-chart.

The study was supervised by an independent scientific committee. The investigators were pharmacists. The pharmacists were recruited from the Celtipharm^®^ panel, which includes 3,004 pharmacies representing the totality of 22,458 pharmacies in continental France, according to the criteria of localization and volume of activity (Fig 2).

Initially, a pre-selection of 700 pharmacies has been done by CELTIPHARM, to propose the study to pharmacies already distributing viscosupplements and other drugs, in relation with knee OA. Following, the first pharmacies having answered positively to CELTIPHARM have been retained to participate to the study. The enrollment was stopped when the sample size of 250 pharmacists, all from continental France was achieved.

The role of the pharmacists involved in this study, was strictly limited to data collection from the patients. The questionnaires submitted to the patients before inclusion, or during the follow-up period have been used as self-questionnaires, meaning that the pharmacists had no influence on the assessment of the patient health (pain, function or Quality of Life).

All the other questions were relative to economic aspects, for which pharmacists played an important role, being at the best place to get the right information for drug delivery, justified by the prescriptions issued from different doctors, as well as other health expenses, related to the knee OA.

Patients received oral and written information on the medico-economic objectives of the study and they signed an informed consent. Patients were allowed to continue their usual treatment and take drugs or consult when needed, in real life conditions, whatever group they were belonging to.

### Ethics statement

As the pharmacist-investigators could not play any intervention role on the treatments, the study was deliberately designed as purely observational and open. The medical doctors—prescribing the treatments—were not participating to the study, and their relations with their patients were totally free, on current practice base. This means that treatments were adapted at any time to the best interest of these patients, without compromise or conflict for the study.

From there and accordingly to the French and other regulations, the study did not have to be registered to health authorities, as pointed out by ICMJE [31].

For the same reason, no approval from an ethics committee was requested, in France. Therefore, no institutional ethics review board, has been solicited for approval.

The database of CELTIPHARM since 2003, received periodically a renewed authorization from the CNIL n°1503551 (decision n° 2011–246 from 08/09/2011), allowing to make studies from anonymous short duration health data.

Regarding confidentiality of data, several levels were in force, through the CELTIPHARM organization:

First, the identification of the questionnaires, was done by the pharmacists at the source, using a strict code format SDDMMYYYYNN, with S for the sex (1 for a man, 2 for a woman), then birthdate (day, month and year, in 8 digits), and first digits for each first name and name.

Second, receipt and control of the data sent by pharmacists, was done at CELTIPHARM by an independent team, in charge of anonymization. Storage was done in a dedicated room, with limited access.

Third, the keyboarding of the questionnaires has been performed by the Direct Marketing Department of CELTIPHARM, in charge of the statistical analysis. The data managers have proceed, to the creation of the input masks, to the quality control and to the data freezing, in accordance with the procedures of CELTIPHARM.

Fourth, only statistical results have been transmitted to the sponsor and authors, who never had access to any individual data, as all these data have been kept inside CELTIPHARM.

Inform consents from the patients, have been obtained through the pharmacists-investigators, at the time of inclusion, and these consents have also been kept inside CELTIPHARM.

### Selection of patients

Pharmacists included patients with the following criteria: man or woman aged between 40 and 75 years; knee OA of radiological known Kellgren-Lawrence (KL) grade II or grade III; symptomatic knee OA requiring NSAIDs at least once a month, since at least 6 months; X-ray report not older than 6 months confirming knee osteoarthritis; WOMAC [32] score (Western Ontario and McMaster Universities) between 30 and 60 (on a 0–100 scale); patient able to understand the requirements of the study and to give informed consent for study participation; patient geographically stable during the study duration. Patients were not included if they had bilateral knee osteoarthritis, infectious or non-infectious inflammatory knee arthritis prior treatment with viscosupplementation or if they were unlikely to understand the conditions for assessment of study criteria or to be followed (Fig 1).

In this prospective study, each pharmacist had to select the first qualifying patients: only one patient under NSAIDs and one patient under IA HA.

### Matching between groups

One critical aspect to be developed is the quality of matching between the two groups, as an important part of data was based on patient declaration, at the time of inclusion.

A good similarity of patient profiles, was first obtained from the strict application of inclusion and non-inclusion criteria. For the IA HA group, the prescription of the viscosupplementation treatment, done by the specialist at month M0, allowed direct pre-selection by the pharmacist. Then for both groups, the patient enrollment was validated by checking that every inclusion and non-inclusion criteria was satisfied (Fig 1), and this inclusion was only confirmed after control by CELTIPHARM.

Retrospective cost analysis was used to confirm the good symmetry of expenses between the two groups, for the M-6 to M0 period: in terms of consultations for each category of practitioner, and in terms of drug consumption.

### Conduct of the study

The study was carried from May 2014 to November 2014, from the first patient enrollment to the last follow-up visit. All enrollments were finalized before June 15, 2014.

During the study, the pharmacist completed seven questionnaires (anonymous case report forms) with the patient (at inclusion and each month during 6 months).

### Evaluation criteria

At each visit, disease status and healthcare resources were recorded. The data collected were demographics, radiological KL grade of knee OA, data from healthcare circuit (treatments delivered by the pharmacist, medical and paramedical consultations, hospitalizations, sick leaves and medical transportation). Only healthcare resources related with knee OA were recorded.

The symptoms of knee OA (WOMAC score) and the quality of life (EQ-5D questionnaire) were assessed at each visit. The WOMAC score consists of 24 items divided into three subscales (pain, 5 items; stiffness, 2 items; physical function, 17 items) [32]. Using the Likert scale, each item was scored from 0 to 4 (0, none; 1, mild; 2, moderate; 3, severe; 4, maximal). The global WOMAC score was calculated by adding the 24 scores and then normalized to give a score from 0 to 100. Each domain (sub-score) was also normalized from 0 to 100. The more the score was high, the more knee osteoarthritis was severe.

The self-administered quality-of-life questionnaire EQ-5D [33] included five questions, each with three levels of response: mobility, self-care, usual activities, pain/discomfort and anxiety/depression. From there, the resulting EQ-5D score was calculated on a 100-base scale: the higher the score, the best the quality of life was, with zero representing the death. As complement, the EQ-VAS (scale 0–100) was also utilized.

### Number of patients to be included

The number of patients to be included in the study was calculated from bilateral test formula

$$\text{N}=2*\left(\sigma^{2}/\Delta^{2}\right)*\left({\text{Z}}_{1-\alpha /2}+{\text{Z}}_{1-\beta }\right)²$$

in which α = 0.05, β = 0.10 (power of the study 90%), Δ = 0.67 from the minimal clinically important difference (improvement) with WOMAC index, and the standard deviation σ = 1.72 obtained for WOMAC index variation from baseline to 3-month follow-up [34].

The result obtained (N = 138), was increased by 40% to anticipate losses to follow-up patients, missing key data, and non-respect of inclusion or non-inclusion criteria. Also, the context of enrollment was narrow in time (6 weeks for the pharmacists, to enroll 2 patients each). Consequently, an enrollment over 200 patients in each group was considered.

### Statistical analysis

Statistical analyzes were performed with software R version 3.0.2. The quantitative variables were described by their mean, standard deviation (SD), and extreme values (minimum and maximum). The qualitative variables were described by the frequency of their modality.

The evaluation of the costs related with knee osteoarthritis were performed from the perspective of the French universal health insurance. The healthcare circuit includes all the following economical items: medical consultations with general practitioners, rheumatologist or other specialists, paramedical consultations, hospitalizations, radiological examinations, drug consumption, devices, stays in healthcare centers, medical transportations and sick leaves. National databases were used for cost evaluation of these different items. Indirect costs were also considered, when resulting from consequences of the disease treatment, as in case of adverse events.

The primary endpoint was the percentage of patients treated with NSAIDs during the follow-up period and the assessment of the impact of IA HA on NSAIDs consumption.

The number of Quality adjusted life year (QALY) was calculated from the differences of EQ-5D scores between groups, weighted by the time spent at health states.

## Results

### Disposition and characteristics of patients

The 252 recruited pharmacists enrolled 429 patients; 28 patients were not analyzed for the following reasons: no transmission of X-rays report, n = 7; not fitting inclusion criteria, n = 5; lost to follow-up, n = 16. Consequently, the analysis population included 401 patients **(**Fig 2**)**.

The mean age of patients was 62.3 years (women 55%) in NSAIDs group and 65.6 years (women 59%) in IA HA group. This difference of age was significant (p < 0.0001), but not considered as critical The radiological grade of knee OA was grade II or III, in same proportion for both groups. At inclusion, the mean scores of WOMAC (three domains and global) were nearly identical (p = 0.75 to 0.95), and it was the same for the quality of life (EQ-5D) mean scores (p = 0.66). Overall, the two NSAIDs and IA HA groups have been considered close together and comparable (Table 1).

**Table 1:** Characteristics of patients at inclusion.

Characteristics of patients	NSAIDs (n = 199)	IA HA (n = 202)	p-value*** (on difference)
Age, years			
Mean (SD)	62.3 (8.2)	65.6 (7.8)	< 0.0001
Min–Max	40–73	42–75	
Sex, n (%)			
Men	89 (45)	83 (41)	
Women	110 (55)	119 (59)	
Radiological stage of knee osteoarthritis, n (%)			
Grade II	103 (52)	109 (54)	
Grade III	96 (48)	93 (46)	
WOMAC score, mean (SD)*			
Pain	50.4 (16.1)	49.9 (17.2)	0.76
Stiffness	45.8 (15.2)	45.7 (15.9)	0.95
Physical function	47.5 (18.7)	48.1 (18.5)	0.75
Global	48.0 (17.9)	48.3 (18.0)	0.77
Quality of life EQ-5D**, mean (SD)*	42 (21)	43 (24)	0.66
NSAIDs uptake (units per month)	2.40	2.02	

* All scores are based on a 0–100 scale

** 5 questions / 3 levels as per EQ-5D-3L

*** Difference was significant for age only, but this was assessed as clinically acceptable, in real life conditions

### Improvement of knee osteoarthritis and quality of life

During the post-inclusion 6 months of the study, the WOMAC score decreased in the three domains (pain, stiffness, physical function) in both groups, thus indicating an improvement of the symptoms of knee OA (Table 2).

**Table 2:** WOMAC and quality of life EQ5D scores.

WOMAC & EQ-5D scores (all in base 100)		NSAIDs (n = 199)			IA HA (n = 202)			Variation* M0 / M3		Variation* M0 / M6		p-value (M3 & M6)
		M0	M3	M6	M0	M3	M6	NSAIDs (n = 199)	IA HA (n = 202)	NSAIDs (n = 199)	IA HA (n = 202)	
WOMAC A, pain (5 questions)	Mean	50.4	46.5	43.5	49.9	33.5	27.6	3.9	16.4	6.9	22.3	
	SD	16.1	17.3	18.1	17.2	17.9	18.2	16.7	17.6	17.1	17.7	
								└────┬────┘		└────┬────┘		
	Difference (SD)							12.5 (17.1)		15.4 (17.4)		< 0.0001
	ES [95% CI]							0.73 [0.53;0.93]		0.88 [0.68;1.08]		
WOMAC B, stiffness (2 questions)	Mean	45.8	43.3	41.1	45.7	31.9	26.2	2.5	13.8	4.7	19.5	
	SD	15.2	16.7	16.6	15.9	17.1	16.8	16.0	16.5	15.9	16.4	
								└────┬────┘		└────┬────┘		
	Difference (SD)							11.3 (16.3)		14.8 (16.1)		< 0.0001
	ES [95% CI]							0.70 [0.50;0.90]		0.92 [0.72;1.12]		
WOMAC C, function (17 questions)	Mean	47.5	45.1	41.8	48.1	38.5	29.3	2.4	9.6	5.7	18.8	
	SD	18.7	19.2	20.1	18.5	20.7	20.2	19.0	19.6	19.4	19.4	
								└────┬────┘		└────┬────┘		
	Difference (SD)							7.2 (19.3)		13.1 (19.4)		< 0.0001
	ES [95% CI]							0.37 [0.17;0.57]		0.68 [0.48;0.88]		
EQ-5D, Quality of Life (5 questions)	Mean	42	51	50	43	56	64	9	13	8	21	
	SD	21	14	22	24	19	16	17.8	21.6	21.6	20.4	
								└────┬────┘		└────┬────┘		
	Difference (SD)							4 (19.9)		13 (21.0)		< 0.0001**
	ES [95% CI]							0.20 [0.002;0.40]		0.62 [0.42;0.82]		
QALY (years) IA HA / NSAIDs	Gain over 3months							0.010		0.032		

* Variations are positive for patient improvement—In the comparison between groups: positive differences are in favor of IA HA

** For EQ-5D in the comparison at M3, p-value = 0.044, which is still significant

**Fig 3:**
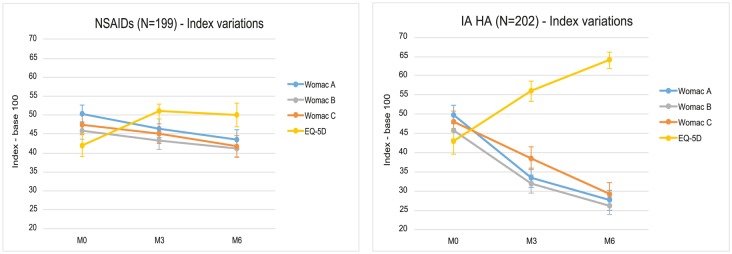
Index variations, for each group NSAIDs or IA HA. Index values for WOMAC A, B, C and for Quality of Life EQ-5D are represented at each observation time, from M0 (inclusion) to M6. All are given in base 100 to allow comparison, and the 95% CI intervals are represented as well. From baseline, a patient improvement is proven by a reduction for any WOMAC index, and by an increase for the EQ-5D. It is obvious that all indexes are clearly improved with IA HA, with a significant difference over NSAIDs.

In the comparisons between groups, all indexes were in favor of IA HA (Fig 3).

The differences were significant (p <0.0001), for all comparisons at 3 and 6 months, with one exception for the Quality of Life EQ-5D index at 3 months, which was significant (p = 0.044), however. This analysis was completed by effect size (ES) evaluation, with 95% CI for the same parameters: at 6 months, ES were found to be > 0.8 (large), for the WOMAC A and B. For the WOMAC C and for EQ-5D, ES was found to be > 0.5 (moderate), all were assessed as good results versus an active comparator.

The EQ-5D score differences at 3 and 6 months, were converted into QALYs attributed to IA HA: 0.01 year from T0 to T3 and 0.032 year from T3 to T6 (Table 2). Over the 6-months follow-up, the gain in QALYs reached by IA HA, was estimated at 0.042 year (0.5 month).

### Medical consultations and radiological examinations

During the 6 months before inclusion, medical (general practitioner, rheumatologist, other specialties) or paramedical (physical medicine, osteopathy) consultations were comparable in the groups NSAIDs and IA HA) (Table 3). After inclusion, there was a significant decrease of the number of consultations to the general practitioner (p < 0.0001) and an increase of visits to the rheumatologist (p = 0.00025) for patients in IA HA group compared to NSAIDs group.

**Table 3:** Medical consultations and radiological examinations before (6 months) and after inclusion (from M0 to M3 and from M4 to M6).

	NSAIDs (n = 199)			IA HA (n = 202)		
	M-6 to M0	M1 to M3	M4 to M6	M-6 to M0	M1 to M3	M4 to M6
**Consultations General practitioner**						
Number of patients	123 (62%)	22 (11%)	35 (18%)	145 (72%)	5 (3%)	9 (5%)
Total number of consultations	296	31	42	309	6	12
***Cost of consultations***	**€6808**	**€713**	**€966**	**€7107**	**€138**	**€276**
***Mean cost per patient***	**€34.21**	**€3.58**	**€4.85**	**€35.18**	**€0.68**	**€1.37**
**Consultations Rheumatologist**						
Number of patients	196 (98%)	56 (28%)	65 (33%)	202(100%)	202(100%)	93 (46%)
Total number of consultations	264	73	82	272	348	103
***Cost of consultations***	**€7392**	**€2044**	**€2296**	**€7616**	**€9744**	**€2884**
***Mean cost per patient***	**€37.15**	**€10.27**	**€11.53**	**€37.70**	**€48.24**	**€14.27**
**Consultations Other specialties**						
Number of patients	38 (19%)	4 (2%)	14 (7%)	41 (20%)	15 (7%)	6 (3%)
Total number of consultations	39	5	16	46	24	9
***Cost of consultations***	**€1326**	**€170**	**€544**	**€1564**	**€816**	**€306**
***Mean cost per patient***	**€6.66**	**€0.85**	**€2.73**	**€7.74**	**€4.04**	**€1.51**
**Paramedical consultations** *						
Number of patients	19 (10%)	14 (7%)	16 (8%)	17 (8%)	6 (3%)	9 (5%)
Total number of consultations	24	16	19	19	7	12
***Cost of consultations***	**€384**	**€256**	**€304**	**€304**	**€112**	**€192**
***Mean cost per patient***	**€1.92**	**€1.29**	**€1.53**	**€1.50**	**€0.55**	**€0.95**
**Radiological examinations**						
Number of patients	199 (100%)	6 (3%)	3 (14%)	202 (100%)	2 (1%)	2 (9.4%)
Total number of examinations	223 (112%)	6 (3%)	3 (16%)	215 (106%)	3 (1%)	2 (10.9%)
***Cost of radiological examinations***	**€6021**	**€135**	**€81**	**€5805**	**€81**	**€54**
***Mean cost per patient***	**€30.25**	**€0.81**	**€0.40**	**€29.17**	**€0.40**	**€0.27**

* Physical medicine/osteopathy.

There was no significant difference between the two groups for the other medical consultations (p = 0.1) and for the paramedical consultations (p = 0.721).

The number of radiological examinations within 6 months before inclusion were comparable in the two groups (Table 3). During the follow-up, there was no significant difference of the two groups radiological examinations (p = 0.668).

### Drug consumption

**Fig 4:**
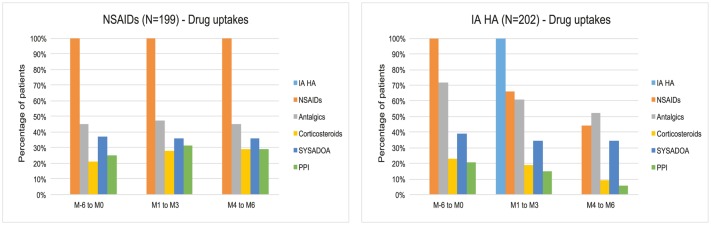
Focus on patients taking drugs. Percentage of each population taking drugs are represented. IA HA treatment (light blue bar) is only present at M1 in the IA HA group. Progressive reduction of population taking NSAIDs, antalgics, corticosteroids and PPI, is important for the IA HA group. Drug consumption is globally stable for the NSAIDs group, with possibly a slight increase for corticosteroids and PPI.

During the 6 months before inclusion, drug consumption was comparable, in quantities and in cost: €98 in NSAIDs group and €101 in IA HA group (Table 4 and Fig 4).

**Table 4:** Drug consumption before inclusion (6 months) and after inclusion (from M1 to M3 and from M4 to M6).

Drugs	NSAIDs (n = 199)			IA HA (n = 202)		
	M-6 to M0	M1 to M3	M4 to M6	M-6 to M0	M1 to M3	M4 to M6
**Arthrum H 2%**						
Number of patients	0 (0%)	0 (0%)	0 (0%)	0	202(100%)	0 (0%)
Mean number of boxes per patient treated	0	0	0	0	1	0
***Mean cost per patient***	**€0**	**€0**	**€0**	**€0**	**€30**	**€0**
**NSAIDs**						
Number of patients	199 (100%)	199 (100%)	199 (100%)	202 (100%)	133 (66%)	89 (44%)
Mean number of boxes per patient treated	14.4	7.3	7.9	12.1	6.4	6.7
***Mean cost per patient***	**€45.36**	**€20.84**	**€22.48**	**€38.18**	**€11.96**	**€8.39**
**Antalgics**						
Number of patients	90 (45%)	94 (47%)	90 (45%)	145 (72%)	123 (61%)	105 (52%)
Mean number of boxes per patient treated	7.2	3.3	3.5	11.4	3.9	3.1
***Mean cost per patient***	**€9.92**	**€4.53**	**€4.94**	**€15.71**	**€5.37**	**€4.26**
**Corticosteroids**						
Number of patients	42 (21%)	56 (28%)	58 (29%)	46 (23%)	38 (19%)	18 (9%)
Mean number of boxes per patient treated	6.6	3.2	3.0	6.3	3.0	3.1
***Mean cost per patient***	**€6.26**	**€3.11**	**€3.07**	**€6.18**	**€3.05**	**€3.09**
**Symptomatic slow action drugs (SYSADOA)**						
Number of patients	74 (37%)	72 (36%)	72 (36%)	79 (39%)	69 (34%)	69 (30%)
Mean number of boxes per patient treated	6.7	3.2	3.2	6.9	3.1	3.0
***Mean cost per patient***	**€27.9**	**€13.6**	**€13.9**	**€28.6**	**€14.9**	**€13.7**
**Proton pump inhibitors (PPI)**						
Number of patients	50 (25%)	62 (31%)	58 (29%)	42 (21%)	30 (15%)	12 (6%)
Mean number of boxes per patient treated	13.4	6.6	6.9	6.0	3.3	3.0
***Mean cost per patient***	**€6.9**	**€5.6**	**€6.1**	**€5.1**	**€3.9**	**€2.9**
**Total for drug treatments**						
***Mean cost per patient***	**€96.34**	**€47.68**	**€50.49**	**€93.77**	**€69.18**	**€32.34**

The expense for NSAIDs treatments significantly decreased in IA HA group (from €38 before inclusion to €20 during follow-up). This result was mainly obtained from patients who discontinued these treatments. In the NSAIDs group, expense for NSAIDs treatments were stable (€45 before inclusion, compared with €43 during follow-up). The NSAIDs individual uptake (average dose per treated patient) was stable in both groups.

A reduction of the uptake of other treatments—antalgics, corticosteroids and proton pump inhibitors (PPI)–was seen for the IA HA group only. In the NSAIDs group, these uptakes remained stable. Before inclusion, the uptake of antalgics was slightly higher in the IA HA group. However, at months M4 to M6, antalgic uptakes were similar between groups.

No difference between groups, was seen for the symptomatic slow action drugs (SYSADOA).

IA HA expenses have been put at M1, as M0 was the month of prescription, this to allow the comparisons before inclusion. As IA HA reimbursement rule accepts 3 injections per knee and per year in France, it was logical to consider 50% of the €60 during the 6-months follow-up period of the study. This reimbursement rule is well applied with Arthrum H 2%, and most patients renew their viscosupplement treatments just after one year [30], or sometimes lately.

Globally, for the follow period (6 months), the expenses for drugs were nearly identical between the 2 groups: €98 for NSAIDs vs €101 for IA HA.

### Hospitalizations and other elements of healthcare circuit

The hospitalizations and other elements of the healthcare circuit that induced a cost for the national health insurance are summarized in Table 5.

**Table 5:** Hospitalizations and other elements of healthcare circuit before (6 months) and after inclusion (from M1 to M3 and from M4 to M6).

	NSAIDs (n = 199)			IA HA (n = 202)		
	M-6 to M0	M1 to M3	M4 to M6	M-6 to M0	M1 to M3	M4 to M6
**Hospitalizations**						
Number of patients	6 (3%)	2 (1%)	3 (1%)	7 (3%)	2 (2%)	0 (0%)
Total number of admissions	8	3	3	7	3	0
***Cost of hospitalizations***	**€4720**	**€1770**	**€1770**	**€4130**	**€1770**	**€0**
***Mean cost per patient***	**€23.71**	**€8.90**	**€8.90**	**€20.44**	**€8.76**	**€0**
**Stays in healthcare center**						
Number of patients	1 (0.5%)	2 (1%)	1 (0.5%)	2 (1%)	0 (0%)	1 (0.5%)
Spa therapy	1	2	1	2	0	1
Rest or retirement home	0	0	0	0	0	0
***Cost of stays in healthcare center***	**€510**	**€1021**	**€510**	**€510**	**€0**	**€510**
***Mean cost per patient***	**€2.57**	**€5.14**	**€2.57**	**€2.57**	**€0**	**€2.57**
**Devices**						
Number of patients	18 (9%)	10 (5%)	12 (6%)	15 (7%)	7 (3%)	5 (2%)
Shoes with flexible sole, knee brace	5	1	1	5	2	2
Walking stick, walker	11	9	11	9	6	3
Wheelchair	2	0	0	1	0	0
***Cost of devices***	**€1073**	**€129**	**€171**	**€656**	**€111**	**€93**
***Mean cost per patient***	**€5.39**	**€0.65**	**€0.86**	**€3.25**	**€0.55**	**€0.46**
**Medical transportations**						
Number of patients	3	2	2	4	2	0
Number of transportations	12.5	1.5	1.5	10	2	0
***Cost of transportations***	**€61.70**	**€49.75**	**€49.76**	**€72.70**	**€56.60**	**€0**
***Mean cost per patient***	**€0.31**	**€0.25**	**€0.25**	**€0.36**	**€0.28**	**€0**
**Sick leaves**						
Number of patients	20	19	21	18	11	8
Percentage of active patients	21%	21%	21%	28%	28%	28%
Total of sick leaves (days)	378	186	198	356	153	107
Mean duration (days)	16	18	17	17	14	11
***Cost of sick leaves***	**€13715**	**€5562**	**€5821**	**€13025**	**€5175**	**€3579**
***Mean cost per patient***	**€68.91**	**€27.95**	**€29.25**	**€64.48**	**€25.62**	**€17.71**

The number of hospitalizations within 6 months before inclusion were comparable in the two groups. During the follow-up, there was a decrease of the cost of hospitalizations, but this difference was not significant (p = 0.44).

There was no significant difference of cost between NSAIDs group and IA HA group for the stays in healthcare centers (p = 0.41), use of devices (p = 0.98), or medical transportations (p = 0.736).

In active patients, a significant decrease of the cost of sick leaves was observed in IA HA group after the first trimester of follow-up compared to NSAIDs group (p = 0.0062).

### Assessment of estimated benefit/risk ratio (primary endpoint)

Before inclusion, 100% of patients were taking NSAIDs in both groups. Comparing the 6-months post IA HA periods (Table 4), the population taking NSAIDs was reduced to 133 patients (66%) at 1–3 months, then to 89 patients (44%) at 4–6 months in the IA HA group, and was kept constant to 199 patients (100%) in the NSAIDs control group.

By significantly reducing the number of patients taking NSAIDs, the local treatment with IA HA, improved the estimated benefit/risk ratio, without compromise for patient health.

### Overall comparison of costs between NSAIDs and IA AH groups

**Fig 5:**
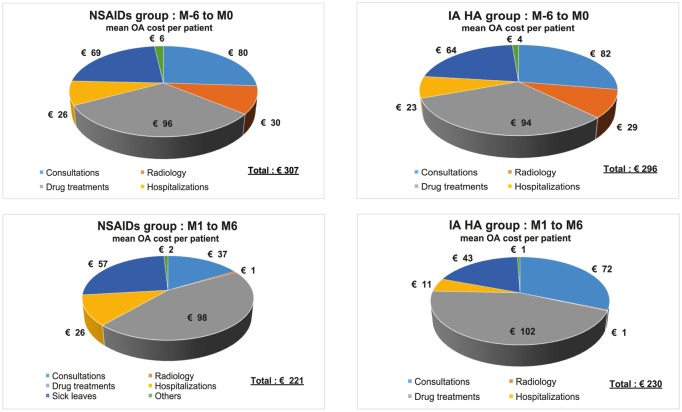
Overview of knee OA related expenses, per average patient, before (M-6 to M0) and after inclusion (M1 to M6). The above 4 graphs, are representing 6-months expenses intervals, with the volume of each pie adjusted to its total expense. The IA HA group M1 to M6 is inclusive of the cost for IA HA treatment. Stays in healthcare centers are grouped with hospitalizations.

There was no significant difference before inclusion for the two groups of patients who were delivered comparable treatments which had similar expenses of €307 or €296 per patient, respectively for the NSAIDs and IAHA groups **(**Table 6 and Fig 5) confirming the good matching between groups. During the follow-up, some costs varied differently according to NSAIDs group and IA HA. For the first 3-month follow up period (M1 to M3) there was a difference of €51 between the two groups, whose an important part was related to the difference of the cost of treatments (NSAIDs or IA HA). For the next 3-month period (M4 to M6), there was a significant decrease of the costs of management in IA HA group. However, the difference between cumulated expenses during the 6-months follow-up period was very small (only €9.03 more for IA HA). As consequence, the global cost during one year (6 months before inclusion and 6 months after) remained quasi identical for NSAIDs group and IA HA group: €528 for NSAIDs vs. €526 for IA HA.

**Table 6:** Overview of expenses of healthcare circuit before inclusion (6 months) and after inclusion (M1 to M3 and M4 to M6).

	NSAIDs (n = 199)			IA HA (n = 202)		
	M-6 to M0	M1 to M3	M4 to M6	M-6 to M0	M1 to M3	M4 to M6
Consultations General practitioner	€34.21	€3.58	€4.85	€35.18	€0.68	€1.37
Consultations Rheumatologist	€37.15	€10.27	€11.53	€37.70	€48.24	€14.27
Consultations Others specialties	€6.66	€0.85	€2.73	€7.74	€4.04	€1.51
Paramedical consultations	€1.92	€1.29	€1.53	€1.50	€0.55	€0.95
Radiological examinations	€30.25	€0.81	€0.40	€29.17	€0.40	€0.27
Drug treatments	€96.34	€47.68	€50.49	€93.77	€69.18	€32.34
Hospitalizations	€23.71	€8.90	€8.90	€20.44	€8.76	€0
Stays in healthcare centers	€2.57	€5.14	€2.57	€2.57	€0	€2.57
Devices	€5.39	€0.65	€0.86	€3.25	€0.55	€0.46
Medical transportation	€0.31	€0.25	€0.25	€0.38	€0.28	€0
Sick leaves	€68.91	€27.95	€29.25	€64.48	€25.62	€17.72
Global mean cost per patient	€307.42	€107.37	€113.36	€296.18	€158.30	€71.46
Total	€528			€526		

As clinical and Quality of Life improvements are obtained at no additional cost for the national health insurance, the cost-utility analysis allows to conclude in favor of IA HA, and satisfy to the second objective of the study. To illustrate, the cost-utility ratio often defined as the cost per QALY, gives €9.03/0.042 = €215 per QALY which is extremely low compared to the willingness-to-pay threshold US$50,000 per QALY [26,27].

## Discussion & conclusion

According to decision tree [20,21] NSAIDs and paracetamol are first-intention treatments prescribed in knee osteoarthritis. NSAIDs may induce important iatrogenic risks [5,7] and paracetamol is known for its relative inefficacy in this disease [6]. It is thus rational to treat locally this disorder, especially in elderly patients who are frequently taking many medications [21].

Medico- or Pharmaco-Economic studies relative to knee OA treatment with IA HA are relatively scarce. We identified several [20–25] and particularly two among them: Kahan [24] and Mazieres [25], both made in the perspective of the French health insurance system.

Despite differences in design, the two studies Kahan and Mazieres reached same conclusion as the present study, to demonstrate that IA HA does not increase expenses in knee OA treatment, and provides benefits to the patients, in terms of clinical results (LEQUESNE and WOMAC indexes) and quality of life SF-12 (Table 7).

**Table 7:** Economic comparison with other studies (base = 6 months periods).

	Kahan (base 1998–2000)		Mazières (base 2003)	
	Control	Synvisc		Suplasn
	Post M1-M6	Post M1-M6	Pre treatment	Post M1-M6
	6 months	6 months	6 months	6 months
Physician visits (all)	€ 68	€ 91	€ 86	€ 42
Other health professionals			*€ 110*	*€ 41*
Examinations (imaging)	€ 21	€ 21	€ 50	€ 11
Drug treatments (w/o IA HA)	€ 181	€ 109	€ 141	€ 113
Medical devices (IA HA)	€ 0	**€ 58**	€ 0	**€ 48**
Hospitalization—Rehabilitation	*€ 255*	*€ 231*	€ 31	€ 48
Non-medical costs	€ 34	€ 29	*€ 201*	*€ 177*
Total cost, per patient	€ 560	€ 540	€ 618	€ 480

Expenses have been re-calculated *prorata temporis* on 6-months periods, to allow comparison with our study.

Higher costs are seen on several items (*in italic characters*), but the differences between groups were relevant.
Hospitalization-rehabilitation, covering all pathologies in Kahan study. Other health professionals: nurses (at home) and therapists, in Mazières study. Non-medical costs: mostly sick leaves, covering all pathologies in Mazières study.

With all observational studies, one main limitation could be the absence of randomization. However, in real life conditions, there was an advantage to keep the patient ignoring the comparison made between treatments. Positively, the relevance of the treatment received was not engaged, minimizing the risk of a bias from patient expectation. Therefore, the comparability of treatment groups at inclusion remains the single critical point which was achieved here, before data interpretation. Moreover, a strong point of this study was the low number of patients lost to follow-up (3.7%; 16/429).

In conclusion, treatment with intra articular hyaluronic acid (Arthrum H 2%), did not generate additional cost for the national health insurance and was associated with a functional improvement of knee OA and quality of life. The economical conditions of 2014, had justified reasonably low expenses for knee OA, starting from €50 per month and patient before treatment, to less than €40 over the 6-months follow-up period of this study. As result, the cost-utility analysis had concluded in favor of hyaluronic acid, based on a better improvement of the pain, function and quality of life (+0.042 QALY), than with the conventional knee OA treatment. In parallel, NSAIDs consumption was significantly decreased (-46.7% in expense) in patients treated with hyaluronic acid, improving the estimated benefit-risk ratio (primary endpoint).

## Supporting information

S1 File Protocol 140330 – Medico eco study ARTHRUM H2%. (PDF)

S2 File QST-LCA 140523-observation TO ARTHRUM H2%. (PDF)

S3 File QST-LCA 140523-observation TO NSAID. (PDF)

S4 File LT-LCA 140523-Kit pharmacists. (PDF)

S5 File trendstatement_trend_checklist 2016-07-12. (PDF)

S6 File DT1-LCA 150318- MEDICO-ECO_Report_MV 04–2016. (PDF)

## Abbreviations

CNIL: Commission nationale de l’informatique et des libertés
EQ-5D: Standardized measure of health status developed by EuroQol Group
ES: Effect size (Cohen’s d)
ESCEO: European society for clinical and economic aspects of osteoporosis and OA
IA HA: Intra-articular hyaluronic acid
ICMJE: International committee of medical journal editors
KL: Kellgren Lawrence (grade)
Mw: Molecular weight (average)
NSAID: Non-steroidal anti-inflammatory drug
OA: Osteoarthritis
PPI: Proton pump inhibitor
QALY: Quality adjusted life year
SOP: Standard operating procedure
SYSADOA: Symptomatic slow acting drug for OA
WOMAC: Western Ontario and McMaster universities (index)
