# Supplementary material for: Intra articular hyaluronic acid in the management of knee osteoarthritis: Pharmaco-economic study from the perspective of the national health insurance system
Source: PLoS One. 2017 Mar 22;12(3):e0173683. doi: 10.1371/journal.pone.0173683 (PMC5362080; doi:10.1371/journal.pone.0173683)
Supplement: S1 File — (PDF) [file pone.0173683.s001.pdf]

MEDICO-ECONOMIC STUDY PROTOCOL

Contact with sponsor

Dr Françoise VINCENT-AUBRY
LCA Pharmaceutical
9 allée Prométhée - ZI Les Propylées
28000 CHARTRES
France
lca@lca-pharma.com

Scientific Committee

Madame Marie-Paule SERRE
Professeur Thierry THOMAS
Monsieur Pierre LEVY
Docteur Françoise AMOUROUX

Contact with supplier of services

Jean-François DERRE
Carré Castan Consultants
141, rue de rennes
75006 PARIS France
jf.derre@ccc.celtipharm.com

Version of protocol & Date 30 March 2014 – Version 3.0

Confidentiality

Information contained in this document is sole property of LCA Pharmaceutical and is transmitted on a confidential base, to competent health authority, investigator, potential investigator, consultant, or member of the Scientific Committee.

None of information contained in this document can be disclosed, except to obtain the consent of persons participating to the study.

Ce document est confidentiel et est la propriété exclusive de CARRE CASTAN CONSULTANTS – groupe CELTIPHARM.
Toute reproduction, diffusion à des tiers est interdite
CELTIPHARM est membre de la Fédération Nationale des Tiers de Confiance

MEDICO-ECONOMIC STUDY PROTOCOL

**Title**	Benefit and utility assessment in knee osteoarthritis care management, in current practice, with intra articular injection of a sodium hyaluronate viscoelastic solution ARTHRUM® H 2%
**Number of version and date**	Version 3.0 dated 2014 / 03 / 30
**Sponsor**	LCA Pharmaceutical
**Study location**	Continental France
**Scientific Committee**	• Madame Marie-Paule SERRE – Professor of Health Marketing, UPMC. Economic Council in Ministry of Health (past) – ENA degree<br>• Professor Thierry THOMAS – Medicine Professor, Rheumatology Department - CHU Saint-Etienne<br>• Monsieur Pierre LEVY – Lecturer - Economy and management of health organizations – Paris Dauphine<br>• Professor Françoise AMOUROUX – Doctor of Pharmacy – Associated Professor – Faculty of Pharmacy Bordeaux
**Investigators**	Pharmacists of dispensaries in continental France
**Objectives**	**Primary Objective : benefit - risk analysis**<br>• To assess the consumption of non-steroidal anti-inflammatory drugs (NSAID)<br>• To assess the iatrogenic risk from the consumption of non-steroidal anti-inflammatory drugs<br>**Secondary Objective : cost - utility analysis**<br>• To assess the treatment costs in current practice, with non-steroidal anti-inflammatory drugs and after or not therapeutic intervention with ARTHRUM® H2%<br>• To assess the Quality of Life of the patient before, and after or not therapeutic intervention with ARTHRUM® H2%, in comparison with current practice with NSAIDs
**Study Design**	Study in real life, longitudinal and multi-centric, comparative between knee osteoarthritis treatment by NSAIDs or by intervention of an intra articular solution, ARTHRUM® H 2%<br><br>**Assessment Methodology** : **benefit-risk and cost-utility analysis**

Ce document est confidentiel et est la propriété exclusive de CARRE CASTAN CONSULTANTS – groupe CELTIPHARM.
Toute reproduction, diffusion à des tiers est interdite
CELTIPHARM est membre de la Fédération Nationale des Tiers de Confiance
CARRE CASTAN CONSULTANTS

**Inclusion Criteria**	• Patient man or woman aged between 40 and 75 years<br>• Patient with knee osteoarthritis Kellgren-Lawrence grade 2 or 3<br>• Patient with symptomatic knee osteoarthritis requiring NSAIDs at least once a month, since at least 6 months<br>• Patient with an X-ray report not older than 6 months confirming knee osteoarthritis<br>• Patient presenting a WOMAC score between 30 and 60, at inclusion<br>• Patient able to understand the requirements of the study and to give informed consent for study participation<br>• Patient geographically stable during the study duration
**Non-inclusion Criteria**	• Patient with bilateral knee osteoarthritis<br>• Patient with infectious or non-infectious knee arthritis<br>• Patient who received a prior treatment with viscosupplementation<br>• Patient unlikely to understand the conditions for assessment of study criteria or to be followed
**Study Procedure**	*Study is managed by a Scientific Committee and by an independent supplier of services*<br><br><u>Recruitment of investigators</u><br>The investigators will be observer pharmacists, enrolled from a representative panel of 3,004 town pharmacies, upon criteria of activity size and location, representing the totality of dispensary pharmacies in France – CELTIPHARM® panel.<br><br>A drawing lots, stratified from the potential of the pharmacies of the panel for the market of knee osteoarthritis, will be made to randomly select 700 pharmacies.<br>Participation to the study will be offered to all selected pharmacies as defined, and the enrollment will be stopped when the sample size of pharmacists accepting to participate will roughly reach the expected target of 250 observer pharmacists, from continental France.<br><br>To warranty the matching between populations, and follow-up of patients during the whole duration of the study, the number of patients included per observer pharmacist will be of maximum two patients (one patient under ARTHRUM® H2%, and one patient under NSAIDs in the context of the knee osteoarthritis).<br><br><u>Selection of patients in real life</u><br>To validate matching of the patients included in the study, investigators must conform to the inclusion criteria. Observer pharmacists have to inform patients on medico- economic objectives of the study, verbally and by written, to get the inform consent form duly signed. Only medico-economic objectives of the study will be described to the patients, to avoid an obvious analysis bias, with a potential Hawthorne effect.<br><br>Following acceptation of the inform consent, matching of populations will be done as follows :<br>The patient will document with help of the pharmacist investigator<br>• Patient profile: age, sex of patient, completed with knee osteoarthritis grade X-ray report.<br>• Use of a decision tree to validate the eligibility of patients included in the study.<br>• WOMAC questionnaire relative to knee osteoarthritis severity: WOMAC score is the most frequently used to assess functional handicap, in patients suffering from knee osteoarthritis.

Ce document est confidentiel et est la propriété exclusive de CARRE CASTAN CONSULTANTS – groupe CELTIPHARM.
Toute reproduction, diffusion à des tiers est interdite
CELTIPHARM est membre de la Fédération Nationale des Tiers de Confiance

This pre-inclusion step will allow pharmacists to include eligible patients and warranty the matching between the groups of population observed.

Each pharmacist investigator will have to include consecutively patients meeting the eligibility criteria of the study and to fill with them the fully anonymous questionnaires, at several times defined in the study schedule.

Moreover, each pharmacist observer will have to complete a non-inclusion register to document the reasons of eligible but non-included patients, over the inclusion period of the study.

Both kits, patient and investigator, will mention a green number, to get any information about the study and its course.

Study course: data collected from patient and pharmacist

- Data collected at inclusion T0 (M0), to allow selection of patient profiles and to validate the patient inclusion:
  - Retrospective analysis over 6 months of the health trajectory
  - WOMAC (Likert scale) and EQ-5D data at T0 (M0)
- Data collected at T1 (M1) / T2 (M2) / T3 (M3) / T4 (M4) / T5 (M5) / T6 (M6):
  - Prospective analysis, every month during 6 months

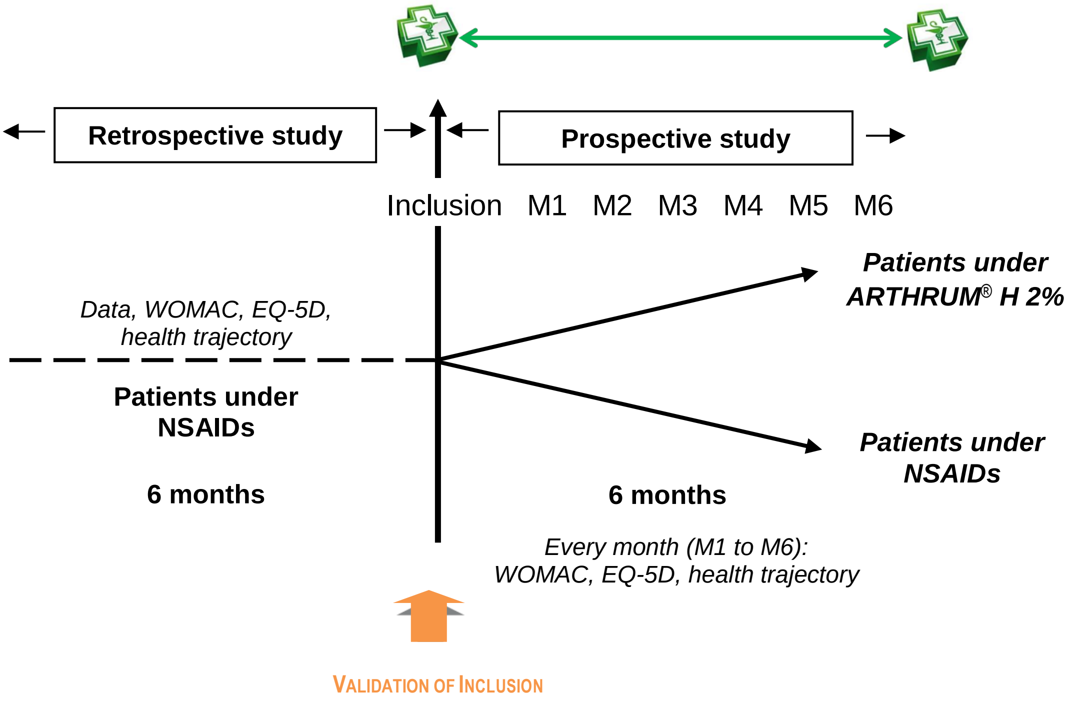

Number of patients necessary

For this study, 2 groups will be analyzed

- Patients treated with NSAIDs then ARTHRUM H2%
- Patients treated with NSAIDs

Calculation of the minimal number of patients necessary: bilateral test

$$N = 2(\sigma^2/\Delta^2)*(Z_{1-\alpha/2}+Z_{1-\beta})^2$$

Ce document est confidentiel et est la propriété exclusive de CARRE CASTAN CONSULTANTS – groupe CELTIPHARM.
Toute reproduction, diffusion à des tiers est interdite
CARRE CASTAN CONSULTANTS
CELTIPHARM est membre de la Fédération Nationale des Tiers de Confiance

	The minimal expected effect, considered as clinically relevant is noted Δ: it is calculated as the mean difference between two treatments.<br>Standard deviation (dispersion) of the main criteria σ → variance σ^2<br><br>The confidence level to be given, to a statistical decision: this is the first kind risk α (often set at 5%), to conclude to a difference which is not existing, between treatments.<br><br>The power of the test (1-β): this is the probability to reject H0 as H0 is wrong (probability to detect an existing difference)<br><br>The number of patients to include is 193 in each arm of the study.<br><br>Finally in the study prospect, 200 patients will be included in each arm ARTHRUM® H2% and NSAIDs.
**Main criteria**	The main criteria of the study will be to measure the percentage of patients treated and the cost of the consumption of non-steroidal anti-inflammatory drugs (NSAIDs) during the follow-up time of the study.<br><br>This criteria will allow to assess in real life, the impact of the therapeutic intervention from intra-articular sodium hyaluronate on the consumption of NSAIDs.
**Data collected**	Data will be collected at several observation times :<br>T0 (M0) / T1 (M1) / T2 (M2) / T3 (M3) / T4 (M4) / T5 (M5) / T6 (M6)<br>Retrospective history upon 6 months, then follow-up during the whole study period (next 6 months)<br><br>To assess the impact of knee osteoarthritis, in real life for a patient, as well as utility of a therapeutic intervention, it is important to get:<br>• An assessment method for health care and investments induced by the pathology<br>• An analytical assessment method including parameters to characterize knee osteoarthritis : pain, stiffness and function<br>• An assessment method for consequences at patient level<br><br>WOMAC osteoarthritis index for lower limbs<br><br>• Pain assessment<br>• Stiffness assessment<br>• Physical function assessment<br><br><u>Health trajectory questionnaire</u><br><br>• Drugs delivered in relation with knee osteoarthritis<br>• Medical and paramedical consultations for outpatient care, in relation with knee osteoarthritis<br>• Hospitalizations and hospital care, in relation with knee osteoarthritis<br>• Sick leaves – Medical transportation – Social services, in relation with knee osteoarthritis<br>• Insurance covered expenses : AMO *(Assurance Maladie Obligatoire)* / AMC *(Assurance Maladie Complémentaire)* and remaining patient part, will be collected<br><br>Drugs delivered in relation with knee osteoarthritis, will be submitted to a consistency checking, with the sale data from the panel Xpr-SO® of CELTIPHARM® as function of date and time of delivery.

Ce document est confidentiel et est la propriété exclusive de CARRE CASTAN CONSULTANTS – groupe CELTIPHARM.
Toute reproduction, diffusion à des tiers est interdite
CELTIPHARM est membre de la Fédération Nationale des Tiers de Confiance

	EQ-5D and QALYs (quality adjusted life years) data<br>• Scores for mobility, self-care, usual activities, pain / discomfort, anxiety / depression<br>• Score for health state<br><br>Register for non-inclusion<br>Sex, Age, WOMAC score at T0, current treatment for knee osteoarthritis, reason (motivation) for non-inclusion.
**Statistic methods**	Sampling method and statistical analysis, will be carried with R software, version 3.0.2<br><br>A sensibility analysis will be done, to analyze the impact of the incoming data, on the variability of output data from the studied model. The sensitivity probabilistic study will be done, using the non-parametric bootstrapping method, applied to individual data, in order to obtain a 95% confidence interval for the cost-utility ratio.<br><br>The 2 following groups treated with NSAIDs (6-months retrospective analysis) will be analyzed for a prospective period of 6 months :<br>• Patients treated with ARTHRUM® H2%<br>• Patients treated with NSAIDs<br><br>Quantitative variables will be described by their mean, median, standard deviation, and extreme values (minimum and maximum). They will be analyzed through the study of differential costs for drug and non-drug expenses, with estimation of a ratio between the 2 groups.<br><br>Qualitative variables will be described by the frequency of each of their modality. The number of QALYs will be calculated by weighting the time spent at health states, by the scores preferably associated with these states. They will be described by studying QALYs differences, with estimation of a ratio between the 2 groups.<br><br>All variables will be checked for their consistency, and verified in order to optimize the quality of data, from a previously validated statistical software.<br><br>Results obtained will be interpreted by the Scientific Committee, for the identification of the main criteria selected.<br><br>An analysis of the register for non-inclusion will allow to verify the representativeness of the patients included in the study.
**Time schedule**	• Final writing of the protocol and questionnaires – validation by the Scientific Committee : Final draft in April 2014<br>• Set up and collect of data : May 2014 to November 2014<br>• Data analysis – Study report : December 2014
**Regulatory authorizations**	• This study does not need to be declared to a CPP *(Comité de Protection des Personnes)* in France.<br>• Declaration to the CNOP *(Conseil National de l'Ordre des Pharmaciens)* for the payment of fees to pharmacists (CELTIPHARM).<br>• Declaration to the CNIL *(Commission Nationale de l'Informatique et des Libertés)* for the protection of personal data :<br>*Authorization CNIL n° 1503551 – Decision n°2011-246 from 08/09/2011 allowing to make studies from anonymous short duration health data.*

Ce document est confidentiel et est la propriété exclusive de CARRE CASTAN CONSULTANTS – groupe CELTIPHARM.
Toute reproduction, diffusion à des tiers est interdite
CELTIPHARM est membre de la Fédération Nationale des Tiers de Confiance
CARRE CASTAN CONSULTANTS fntc AFCDP
