# Supplementary material for: Intra articular hyaluronic acid in the management of knee osteoarthritis: Pharmaco-economic study from the perspective of the national health insurance system
Source: PLoS One. 2017 Mar 22;12(3):e0173683. doi: 10.1371/journal.pone.0173683 (PMC5362080; doi:10.1371/journal.pone.0173683)
Supplement: S3 File — (PDF) [file pone.0173683.s003.pdf]

Questionnaire for your patient under NSAIDs

Medico-economical study • Questionnaire TO - N°1 / 7

Part I	*WOMAC*	Date of control: / / 2014
Part II	*Health care trajectory*	Patient code[1]: _
Part III	*Quality of Life*	Investigator name initials: _
		Personalized survey number: "CTPM_IDPHARMA"

PATIENT PROFILE

X-RAY REPORT

Date of report / / 2014

Knee osteoarthritis grade

001. □ Grade 0: normal
002. □ Grade 1: possible osteophytic lipping
003. □ Grade 2: definite osteophytes and doubtful joint space narrowing
004. □ Grade 3: multiple osteophytes and definite joint space narrowing
005. □ Grade 4: marked pinched joint space narrowing and sub-chondral bone sclerosis

SEX

001. □ Man
002. □ Woman

AGE *(mention the right age and nick the corresponding age range)*

/__ /year

001. □ from 40 to 44 years
002. □ from 45 to 49 years
003. □ from 50 to 54 years
004. □ from 55 to 59 years
005. □ from 60 to 64 years
006. □ from 65 to 69 years
007. □ from 70 to 75 years

[1] This code is set from:

1. One number indicating the sex (1 for a man and 2 for a woman)
2. Birthday at format DDMMYYYY
3. First letter of first name and first letter of name

Ex: For Thomas DUPONT born on 01/01/1970, patient's code is: 101011970TD

Decision tree to validate eligibility criteria for patient inclusion in the observatory

To be filled by the health professional, with patient's contribution

1	Is the patient aged from 40 to 75 years?	YES<br>Next question	NO<br>STOP INCLUSION
2	Is the patient suffering from a grade 2 or 3, symptomatic knee osteoarthritis?	YES<br>Next question	NO<br>STOP INCLUSION
3	Is the patient affected by a bilateral knee osteoarthritis?	NO<br>Next question	YES<br>STOP INCLUSION
4	Is the patient affected by an infectious or non-infectious knee arthritis?	NO<br>Next question	YES<br>STOP INCLUSION
5	Did the patient receive previously a viscosupplementation treatment?	NO<br>Next question	YES<br>STOP INCLUSION
6	Did the patient take NSAIDs at least once a month during the last 6 months?	YES<br>Next question	NO<br>STOP INCLUSION
7	Is the patient able to provide an X-ray report dated from less than 6 months, confirming radiological grade of the knee osteoarthritis?	YES<br>Next question	NO<br>STOP INCLUSION
8	Does the patient have a WOMAC score at TO, between 30 and 60?	YES<br>Next question	NO<br>STOP INCLUSION
9	Is the patient able to understand how the study takes place?	YES<br>Next question	NO<br>STOP INCLUSION
10	Is the patient able to give his written consent to participate to the study?	YES<br>Next question	NO<br>STOP INCLUSION
11	Is the patient geographically stable during the whole study?	YES<br>Next question	NO<br>STOP INCLUSION

** Positive inclusion to participate to the observatory, you can start with the questionnaire to the patient*

Part I • WOMAC®

WOMAC® OSTEOARTHRITIS INDEX

For each of the domains, qualify[2] the severity of each following item: pain, stiffness and physical function among 5 possible responses: none, mild, moderate, severe or extreme

	None = 0	Mild = 1	Moderate = 2	Severe = 3	Extreme = 4
**Pain domain: how important is your pain?**					
1 – walking on a flat surface?					
2 – going up or down stairs?					
3 – at night, while in bed?					
4 – sitting on, or standing from a chair?					
5 – standing upright?					
**Stiffness domain: how severe is the stiffness of your joint?**					
1 – after awaking on morning?					
2 – after sitting, lying or resting later in the day?					
**Physical function domain: how important are your difficulties for?**					
1 – descending (going down) stairs?					
2 – ascending (going up) stairs?					
3 – rising from sitting?					
4 – standing?					
5 – bending to floor?					
6 – walking on a flat surface?					
7 – getting in / out of car?					
8 – going shopping?					
9 – putting on socks / stockings?					
10 – rising from bed?					
11 – taking off socks / stockings?					
12 – lying in bed?					
13 – getting in / out of bath?					
14 – sitting?					
15 – getting on / off toilet?					
16 – heavy domestic duties: doing the housework from top to bottom?					
17 – light domestic duties: tidying, dusting, cooking?					
**TOTAL SCORE**					

Source: Bellamy N, Buchanan WW, Goldsmith CH, Campbell J, Stit LWJ. Validation of WOMAC: a health status instrument for measuring clinically important patient relevant outcomes to antirheumatic drug therapy in patients with osteoarthritis of the hip or knee. J Rheumatol 1995; 15: 1833-40

(2) Qualification of the table:

1. The patient must answer to each question accordingly to a quotation from 0 to 4 in corresponding column, among five possible choices
2. The total score is obtained by adding the results to each question

Part II• Health care trajectory of the patient

A. Hospitalization in relation with knee osteoarthritis during the last 6 months

1. Have you been hospitalized (one or several nights at hospital) for your knee osteoarthritis, during the last 6 months?

☐ Yes *(continue with question 1a)*
☐ No *(go to question 2)*

1a. If yes, indicate the dates and the hospital department, where you have been hospitalized for your knee osteoarthritis, during the last 6 months.

	Department	Date of arrival	Date of exit	Medical care
Admittance 1		...../ ./.......	./ ./	
Admittance 2		./ ./.......	./ ./	
Admittance 3		...../ ./.......	./ ./	
Admittance 4		...../ ./.......	./ ./	
Admittance 5		...../ ./.......	./ ./	

2. In relation with your knee osteoarthritis, did you receive one or several outpatient operations (arrival and exit the same day from the health facility), during the last 6 months?

☐ Yes *(continue with question 2a)*
☐ No *(go to question 3)*

2a. If yes, which medical care did you receive?:

	Type of medical care
Intervention 1	
Intervention 2	
Intervention 3	
Intervention 4	
Intervention 5	

3. How many X-ray examinations did you receive, in relation with knee osteoarthritis during the last 6 months?

Number of X-ray exams: ______________________

B. Medical consultations in relation with knee osteoarthritis during the last 6 months

4. In relation with your knee osteoarthritis, did you consult physicians and/or nurses during the last 6 months?

☐ Yes *(continue with question 4a)*
☐ No *(go to question 5)*

4a. If YES to question 4, indicate for each health professional, the specialty, the way of practice and the number of consultations.

Health professional # 1	Specialty<br>*(1 answer only allowed)*<br>☐ general practitioner<br>☐ rheumatologist<br>☐ nurse<br>☐ other : specify	Way of practice<br>*(1 answer only allowed)*<br>☐ private practice<br>☐ hospital<br>☐ clinic<br>☐ mixt : private / salaried<br>☐ other : specify	*Number of consultations in practice location :*<br>*Number of consultations at home :*
Health professional # 2	Specialty<br>*(1 answer only allowed)*<br>☐ general practitioner<br>☐ rheumatologist<br>☐ nurse<br>☐ other : specify	Way of practice<br>*(1 answer only allowed)*<br>☐ private practice<br>☐ hospital<br>☐ clinic<br>☐ mixt : private / salaried<br>☐ other : specify	*Number of consultations in practice location :*<br>*Number of consultations at home :*
Health professional # 3	Specialty<br>*(1 answer only allowed)*<br>☐ general practitioner<br>☐ rheumatologist<br>☐ nurse<br>☐ other : specify	Way of practice<br>*(1 answer only allowed)*<br>☐ private practice<br>☐ hospital<br>☐ clinic<br>☐ mixt : private / salaried<br>☐ other : specify	*Number of consultations in practice location :*<br>*Number of consultations at home :*
Health professional # 4	Specialty<br>*(1 answer only allowed)*<br>☐ general practitioner<br>☐ rheumatologist<br>☐ nurse<br>☐ other : specify	Way of practice<br>*(1 answer only allowed)*<br>☐ private practice<br>☐ hospital<br>☐ clinic<br>☐ mixt : private / salaried<br>☐ other : specify	*Number of consultations in practice location :*<br>*Number of consultations at home :*
Health professional # 5	Specialty<br>*(1 answer only allowed)*<br>☐ general practitioner<br>☐ rheumatologist<br>☐ nurse<br>☐ other : specify	Way of practice<br>*(1 answer only allowed)*<br>☐ private practice<br>☐ hospital<br>☐ clinic<br>☐ mixt : private / salaried<br>☐ other : specify	*Number of consultations in practice location :*<br>*Number of consultations at home :*

Other paramedical consultations - Ex: physical therapist

5. In relation with your knee osteoarthritis, did you need further health care during the last 6 months, with intervention of other health professionals ?
For instance, as a physical therapist

☐ Yes *(continue with question 5a)*
☐ No *(go to question 6)*

5a. If YES to question 5, indicate the number and the way of consultations, performed with these health professionals during the last 6n months.

Consultations	*Nb consultations at private practice location, during last 6 months*	*Nb consultations at home during last 6 months*
Physical therapist		
Osteopath		
Acupuncturist		
Other		

<u>Phone consultations following knee osteoarthritis</u>

6. In relation with your knee osteoarthritis, did you use consultations by phone with health professionals, during the last 6 months?

- ☐ Yes *(continue with question 6a)*
- ☐ No *(go to question 7)*

6a. If YES to question 6, how many phone calls did you make for this matter, during the last 6 months?

Also indicate which health professionals were giving their advice.

Advice given by	*Nb of phone consultations during last 6 months*
General practitioner	
Nurse	
Rheumatologist	
Other (specify):	

C. <u>Drugs</u> *(To be filled together between patient and pharmacist, using the history of deliveries)*

7. What are all the drugs taken by your patient during the last 6 months, to treat his knee osteoarthritis?

Treatments	*Dates of delivery*	*Hours of uptake*	*Drugs taken on regular base*	*Drugs taken on patient initiative*
	...../...../........	_ _ : _ _		
	...../...../........	_ _ : _ _		
	...../...../........	_ _ : _ _		
	...../...../........	_ _ : _ _		
	...../...../........	_ _ : _ _		
	...../...../........	_ _ : _ _		
	...../...../........	_ _ : _ _		
	...../...../........	_ _ : _ _		
	...../...../........	_ _ : _ _		
	...../...../........	_ _ : _ _		
	...../...../........	_ _ : _ _		
	...../...../........	_ _ : _ _		

For information, non-exhaustive list of specialty classes often prescribed in knee osteoarthritis:

- *Per os analgesics*
- *Local analgesics*
- *Psychotropic drug*
- *Hormone replacement therapy*
- *Dietary supplements*
- *Vitamins and mineral salts*

D. <u>Medical devices and life help equipment</u> *(To be filled together between patient and pharmacist)*

8. In relation with your knee osteoarthritis, did you use medical devices or life help equipment, during the last 6 months?

☐ Yes *(continue with question 8a)*
☐ No *(go to question 9)*

8a. If YES to question 8, indicate the type of medical devices or life help equipment, the total cost of it and the remaining charge to the patient.

Medical devices and life help equipment	*Renting (YES / NO)*	*Cost in €*	*Remaining charge to the patient in €*
Soft sole shoes	☒		
Orthosis adapted to the knee	☒		
Use of a stick			
Aid with a walking frame			
Elevator for person with reduced mobility - lift			
Medical bed			
Wheelchair			
Other (specify) :			
Other (specify) :			

E. <u>Social services and others, in relation with knee osteoarthritis</u>

9. During the last 6 months, did you stay in a rest home, a retirement home, for a water cure or in another housing structure, in relation with the care of your knee osteoarthritis?

☐ Yes *(continue with question 9a)*
☐ No *(go to question 10)*

9a. If YES to question 9, how many days did you stay in these structures during the last 6 months ?

Housing	*Nb of days, during last 6 months*	*Cost in €*	*Remaining charge to the patient in €*
Water cure			
Rest home			
Retirement home			
Other kind :			
Other kind :			
Other kind :			

10. In relation with your knee osteoarthritis care, did you need home help services, during the last 6 months?

☐ Yes *(continue with question 10a)*
☐ No *(go to question 11)*

10a. If YES to question 10, indicate the number of hours per months and the number of months

____ Hours / month, over ________ months

11. In relation with your knee osteoarthritis care, did you need to use medical transportation, during the last 6 months?

☐ Yes *(continue with question 11a)*
☐ No *(go to question 12)*

11a. If YES to question 11, indicate the number of hours per month and the number of months concerned

Transportation	***Nb of months***	***Nb of hours***	*Remaining charge to the patient in €*
Individual transport			
Public transport			
Approved taxis for sick people			
Ambulance transport			
Other transport :			
Other transport :			

F. <u>Questionsrelative to employment</u>

12. What is your actual professional situation ?

☐ Active (salaried, employee, liberal profession…) *(continue with questions 12a and 12b)*

☐ Retired
☐ Invalid
☐ Un-employed
☐ Non- active
☐ Other _

(go to question 13)

12a. Do you work at :

☐ Full time
☐ Partial time: Equivalent percentage to full time: _______%

12b. In relation with your knee osteoarthritis, how many days of did you get for sick leave, during the last 6 months?

Number of sick leave days: _

G. <u>Investments and help tools</u>

13. During the last 6 months, did you make works to modify your home or your car, or did you need specific equipments or tool helps to overcome the handicap, following your knee osteoarthritis ?

☐ Yes *(continue with question 13a)*
☐ No *(go to Part III)*

13a. If YES to question 13, type of investments, total cost of them and remaining share to the patient.

	Total cost in €	*Remaining charge to the patient in €*
Elevator for person with reduced mobility - lift		
Ramps, bars, handrails		
Other modifications to your home (kitchen, bathroom, bed, alarm…)		
Tools and special aids (to write, to make cooking, for personal hygiene, to dress oneself…)		
Other :		

Part III • Quality of life

To be filled by the patient

By placing a tick in one box in each group below, please indicate which statements best describe your own health state today.

Mobility
001. I have no problem in walking about ☐
002. I have some problems in walking about ☐
003. I am confined to bed ☐

Self-Care
001. I have no problem with self-care ☐
002. I have some problems washing or dressing my-self ☐
003. I am unable to wash or dress myself ☐

Usual activities *(e.g. work, study, housework, family or leisure activities)*
001. I have no problem with performing my usual activities ☐
002. I have some problems with performing my usual activities ☐
003. I am unable to perform my usual activities ☐

Pain / Discomfort
001. I have no pain or discomfort ☐
002. I have moderate pain or discomfort ☐
003. I have extreme pain or discomfort ☐

Anxiety / Depression
001. I am not anxious or depressed ☐
002. I am moderately anxious or depressed ☐
003. I am extremely anxious or depressed ☐

How would you qualify your health state today, on a scale 0 to 100?
Indicate your answer and tick the corresponding box.

0 (Worst imaginable health state) and 100 (Best imaginable health state):

______ / Health state

001. ☐ From 01 to 10
002. ☐ From 11 to 20
003. ☐ From 21 to 30
004. ☐ From 31 to 40
005. ☐ From 41 to 50
006. ☐ From 51 to 60
007. ☐ From 61 to 70
008. ☐ From 71 to 80
009. ☐ From 81 to 90
010. ☐ From 91 to 100

End of visit – We answer you for your participation -
The next questionnaire to be filled with your patient will be T1 N° 2 / 7
