# Supplementary material for: Intra articular hyaluronic acid in the management of knee osteoarthritis: Pharmaco-economic study from the perspective of the national health insurance system
Source: PLoS One. 2017 Mar 22;12(3):e0173683. doi: 10.1371/journal.pone.0173683 (PMC5362080; doi:10.1371/journal.pone.0173683)
Supplement: S4 File — (PDF) [file pone.0173683.s004.pdf]

National Observatory for the treatment with NSAIDs and ARTHRUM H 2% in knee osteoarthritis

«CTPM_INDEX»-«CTPM_IDPHARMA»
«CTPM_NOMUSAGE»
«CTPM_CIVTIT1 » «CTPM_PNOMTIT1 » «CTPM_NOMTIT1 »
«CTPM_ADR1 »
«CTPM_ADR2»
«CTPM_ADR3»
«CTPM_CP» «CTPM_VILLE»

We thank you for your acceptance to participate to the study named « *National Observatory for the treatment with NSAIDs and ARTHRUM H2% in knee osteoarthritis* », **This study will be carried from May 2014 to November 2014 and the selected patients have to remain the same, during the whole follow-up period.**

Please find herewith:

- An instruction giving details for how to proceed
- Eligibility for patients, and sub-groups of patients
- Decision tree to classify the patients to be included in the Observatory
- A calendar for the follow-up of the study
- The non-inclusion register, to be returned by fax
- An acknowledgment form, to be returned by fax

→ **Green N° : 0805 02 13 35**

- **The questionnaire n°** 1 **/ 7** in double sample (for your patients under *NSAIDs and ARTHRUM H2%)* to be returned once data collected, **before June 15, 2014, by the way of your choice** underneath:
    1. by using one of the T envelopes (enclosed)
    2. by fax to: → **Green N° : 0805 02 13 35**
    3. by email to: ml-ctpm@celtipharm.com, with the mention « *National Observatory for knee osteoarthritis »*

The questionnaires n° 2 to 7 will sent to you, by the middle of the month, to the e-mail address, that you have been giving to us by phone.
We are at your disposal for any complement information, and you can contact us at:

N° Vert 0805 02 13 34

L'institut CTPM

Celtipharm
pour une Santé Raisonnée®

National Observatory for the treatment with NSAIDs and ARTHRUM H 2% in knee osteoarthritis

To be kept

Instructions for study management

Name of the person in charge, in the pharmacy (drugstore):

Name of the person in charge, in the pharmacy (drugstore), in case of vacation:

1- □ Upon receipt of this participation kit, thank you to send back the acknowledgment form (page 7)
2- □ Have the non-inclusion register available at the desk (page 5)
3- □ Ask the questions for inclusion and:
- recruit the corresponding patients
- note the eligible – but not recruited – patients, in the register

4- □ Once the corresponding patients recruited, fill information in the questionnaires, with the patient under NSAIDs, and with the patient under ARTHRUM H 2%
5- □ Using your follow-up calendar (page 6), fix the appointment dates for the next 6 months with these same patients (also indicate the corresponding patient code*)
6- □ Return the questionnaires to us by the channel of your choice

Documents to return :

Non-inclusion register
X-ray report
Acknowledgment for the kit
Monthly questionnaires for the 2 types of treatment

DOCUMENTS	STATUS	DATE
Non-inclusion register	□ Done	..... ./..... ./........
X-ray report	□ Done	..... ./..... ./........
Acknowledgment for the kit	□ Done	....../..... ./........
Monthly questionnaires for the 2 types of treatment	□ Done	*Refer to the follow-up calendar*

Remind of instructions for return:

- Use one of the T envelopes (enclosed) – or postal address: INSTITUT CTPM - CTPM ROUTAGE-AUTORISATION 10124-56029 VANNES CEDEX (free mailing)
- Fax to: **→ Green N° : 0805 02 13 35**
- Email to: ml-ctpm@celtipharm.com, with the mention « *National Observatory for knee osteoarthritis* »

*This patient code is formed by:

1. One number for the sex: 1 for a man, and 2 for a woman)
2. Birth date in format: DDMMYYYY
3. First letter of the first name followed by first letter of the name

Ex: For Thomas DUPONT, born on 01/01/1970, the patient code will be: 101011970TD

National Observatory for the treatment with NSAIDs and ARTHRUM H 2% in knee osteoarthritis

To be kept

<u>Patient eligibility criteria</u>

<u>Inclusion criteria</u>:

- Patient male or female, from 40 to 75 years old
- Patient with symptomatic knee osteoarthritis grade 2 or grade 3
- Patient with symptomatic knee osteoarthritis, justifying uptake of NSAIDs at least once a month since over 6 months
- Patient with W-ray report dated from less than 6 months, confirming a knee osteoarthritis
- Patient with a WOMAC score from 30 to 60, at inclusion
- Patient able to understand the study course and to give is written consent for his participation
- Patient geographically stable during the totality of the study

<u>Non-inclusion criteria</u>:

- Patient with a bilateral knee osteoarthritis (OA)
- Patient with an inflammatory arthritis of the knee, infectious or non-infectious
- Patient having anteriorly received a visco-supplementation treatment
- Patient liable not to understand the assessment procedure for the study criteria, or not to come to follow-up visits

<u>Groups of patients included</u>

Only the 2 following OA patients groups will be included:

- Patients with knee OA under NSAIDs at time of inclusion
- Patients with knee OA with ARTHRUM H 2% prescribed just at the time of inclusion

Patients of both groups, have all been treated with NSAIDs at least for 6 months before inclusion.

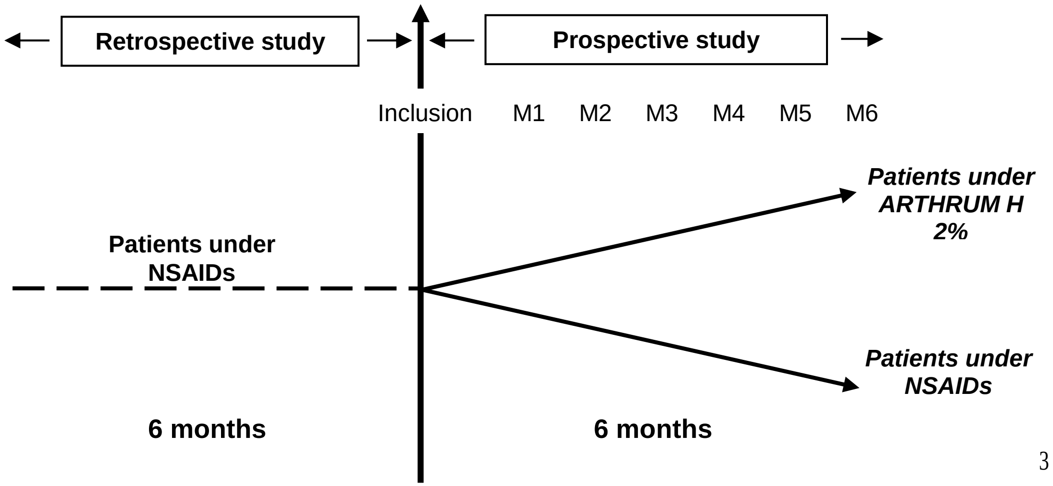

National Observatory for the treatment with NSAIDs and ARTHRUM H 2% in knee osteoarthritis

To be kept

<u>Decision tree for the classification of patients selected to the observatory</u>

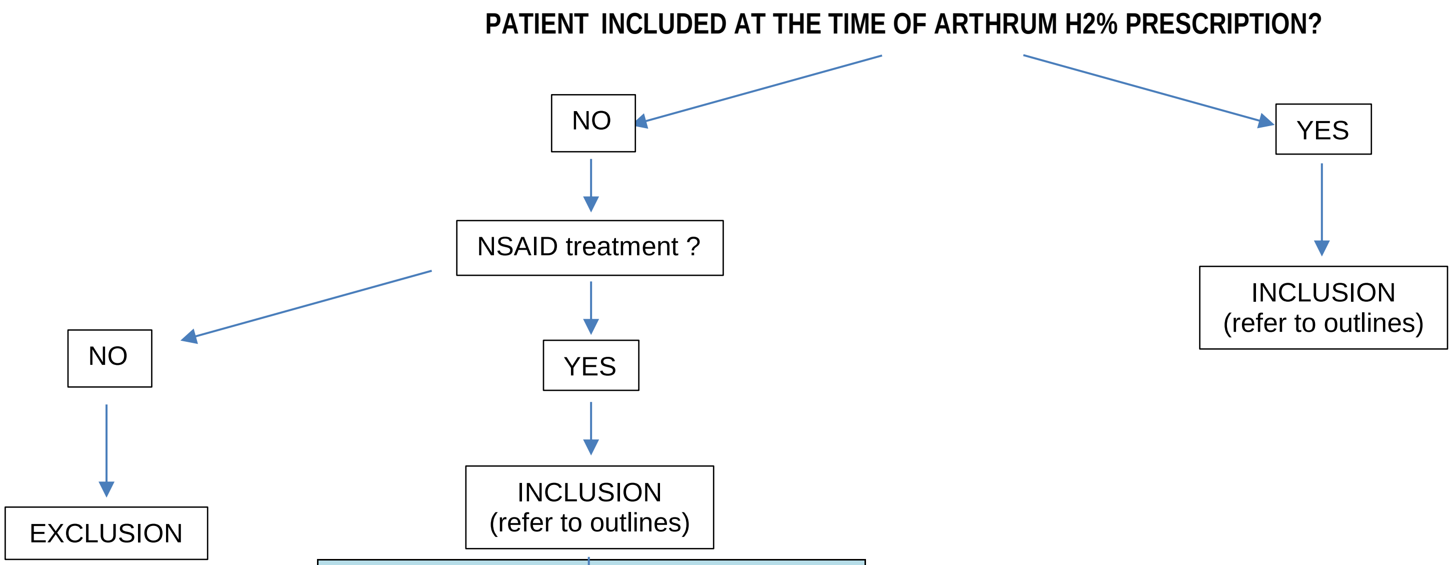

Patients under NSAIDs, if one of the following products is used for OA treatment	
Class - Treatment	
ACECLOFENAC	TIAPROFENIC ACID
NEFAMIC ACID	ALMINOPROFEN
NIFLURIMIC ACID	CELECOXIB
DICLOFENAC	ETODOLAC
FENOPROFEN	FLURBIPROFEN
IBUPROFEN	INDOMETACINE
KETOPROFEN	MELOXICAM
NABUMETONE	NAPROXEN
PIROXICAM	SUDINLAC
TENOXICAM	

National Observatory for the treatment with NSAIDs and ARTHRUM H 2% in knee osteoarthritis

To be returned

Non-inclusion register : *Please note in this register all patients eligible, but not included (1 line/patient)*

TO BE COMPLETED AND RETURNED BY FAX AT: → **Green N° : 0805 02 13 35**

Line N°	Sex	Age	WOMAC score at T0	Present treatment	Non-inclusion reason	
1	□ Man<br>□ Woman	|__|__| years	|__|__|	□ NSAIDs<br>□ ARTHRUM H 2%<br>□ Other:_______________	□ Declined to participate<br>□ Speaks/reads badly French<br>□ Other:_______________________	□ Anterior visco-supplementation treatment<br>□ Patient with infectious arthritis<br>□ Other:_______________________
2	□ Man<br>□ Woman	|__|__| years	|__|__|	□ NSAIDs<br>□ ARTHRUM H 2%<br>□ Other:_______________	□ Declined to participate<br>□ Speaks/reads badly French<br>□ Other:_______________________	□ Anterior visco-supplementation treatment<br>□ Patient with infectious arthritis<br>□ Other:_______________________
3	□ Man<br>□ Woman	|__|__| years	|__|__|	□ NSAIDs<br>□ ARTHRUM H 2%<br>□ Other:_______________	□ Declined to participate<br>□ Speaks/reads badly French<br>□ Other:_______________________	□ Anterior visco-supplementation treatment<br>□ Patient with infectious arthritis<br>□ Other:_______________________
4	□ Man<br>□ Woman	|__|__| years	|__|__|	□ NSAIDs<br>□ ARTHRUM H 2%<br>□ Other:_______________	□ Declined to participate<br>□ Speaks/reads badly French<br>□ Other:_______________________	□ Anterior visco-supplementation treatment<br>□ Patient with infectious arthritis<br>□ Other:_______________________
5	□ Man<br>□ Woman	|__|__| years	|__|__|	□ NSAIDs<br>□ ARTHRUM H 2%<br>□ Other:_______________	□ Declined to participate<br>□ Speaks/reads badly French<br>□ Other:_______________________	□ Anterior visco-supplementation treatment<br>□ Patient with infectious arthritis<br>□ Other:_______________________
6	□ Man<br>□ Woman	|__|__| years	|__|__|	□ NSAIDs<br>□ ARTHRUM H 2%<br>□ Other:_______________	□ Declined to participate<br>□ Speaks/reads badly French<br>□ Other:_______________________	□ Anterior visco-supplementation treatment<br>□ Patient with infectious arthritis<br>□ Other:_______________________
7	□ Man<br>□ Woman	|__|__| years	|__|__|	□ NSAIDs<br>□ ARTHRUM H 2%<br>□ Other:_______________	□ Declined to participate<br>□ Speaks/reads badly French<br>□ Other:_______________________	□ Anterior visco-supplementation treatment<br>□ Patient with infectious arthritis<br>□ Other:_______________________
8	□ Man<br>□ Woman	|__|__| years	|__|__|	□ NSAIDs<br>□ ARTHRUM H 2%<br>□ Other:_______________	□ Declined to participate<br>□ Speaks/reads badly French<br>□ Other:_______________________	□ Anterior visco-supplementation treatment<br>□ Patient with infectious arthritis<br>□ Other:_______________________
9	□ Man<br>□ Woman	|__|__| years	|__|__|	□ NSAIDs<br>□ ARTHRUM H 2%<br>□ Other:_______________	□ Declined to participate<br>□ Speaks/reads badly French<br>□ Other:_______________________	□ Anterior visco-supplementation treatment<br>□ Patient with infectious arthritis<br>□ Other:_______________________
10	□ Man<br>□ Woman	|__|__| years	|__|__|	□ NSAIDs<br>□ ARTHRUM H 2%<br>□ Other:_______________	□ Declined to participate<br>□ Speaks/reads badly French<br>□ Other:_______________________	□ Anterior visco-supplementation treatment<br>□ Patient with infectious arthritis<br>□ Other:_______________________

National Observatory for the treatment with NSAIDs and ARTHRUM H 2% in knee osteoarthritis

To be kept

Calendar for study follow-up

From May 2014 to November 2014

	May	June	July	August	September	October	November
N° of questionnaire	**T0 – 1/7**	**T1 – 2/7**	**T2 – 3/7**	**T3 – 4/7**	**T4 – 5/7**	**T5 – 6/7**	**T6 – 7/7**
Date of return							
Visit dates for your patient under NSAIDs							
NSAIDS patient's code*							
Visit dates for your patient under ARTHRUM H 2%							
ARTHRUM H 2% patient's code*							

*This patient code is formed by:

1. One number for the sex: 1 for a man, and 2 for a woman)
2. Birth date in format: DDMMYYYY
3. First letter of the first name followed by first letter of the name

Ex: For Thomas DUPONT, born on 01/01/1970, the patient code will be: 101011970TD

National Observatory for the treatment with NSAIDs and ARTHRUM H 2% in knee osteoarthritis

To be returned

«CTPM_INDEX»-«CTPM_IDPHARMA»
«CTPM_NOMUSAGE»
«CTPM_CIVTIT1» «CTPM_PNOMTIT1»«CTPM_NOMTIT1»
«CTPM_ADR1»
«CTPM_ADR2»
«CTPM_ADR3»
«CTPM_CP»«CTPM_VILLE»

Vannes, le 23/05/2014

OBJECT: ACKNOWLEDGMENT
Ref: 008-MD-LCA-140506-KIT PARTICIPATION COURRIER_FGO

Please find herewith, accordingly to our general conditions for services:

Parcel	Content	Carrier
1 envelope	1 participation kit, to the study	La Poste

ACKNOWLEDGMENT

Thank you to send it back by fax at: → Green N° : 0805 02 13 35

I, undersigned,

First name: Name:

Confirm to have received the parcel, whose content is in conformity with the description given in the enclosed letter and engage myself to participate to the study until its end.

Date: _

Signature:

Stamp of the pharmacy
