# Supplementary material for: Intra articular hyaluronic acid in the management of knee osteoarthritis: Pharmaco-economic study from the perspective of the national health insurance system
Source: PLoS One. 2017 Mar 22;12(3):e0173683. doi: 10.1371/journal.pone.0173683 (PMC5362080; doi:10.1371/journal.pone.0173683)
Supplement: S6 File — (PDF) [file pone.0173683.s006.pdf]

MEDICO-ECONOMICAL STUDY ARTHRUM H 2%
Final Report

Report made for LCA

Contact :
Mme VINCENT-AUBRY, Directrice Général
LCA Pharmaceutical
9 allée Prométhée - ZI Les Propylées
28000 CHARTRES
France
lca@lca-pharma.com

Date : 18/03/2015

Confidentialité

Les informations contenues dans ce document sont la propriété de LCA Pharmaceutical et vous sont transmises confidentiellement en tant qu'autorité de santé compétente, investigateur, investigateur potentiel, consultant, ou membre du comité scientifique.

Aucune des informations contenues dans ce document ne doivent être divulguées, excepté pour l'obtention du consentement des personnes qui participeront à l'étude.

Contents

1 - Objectives and study design 4

A. Objectives of the study 4

B. Design of the study 5

2 - Population and enrollment 6

A. Recruitment of investigators 6

B. Enrollment of patients in real life 6

3 - Data collection 9

A. Population: method for collecting data 9

B. Identification data for the population 9

C. Collected variables 9

1. WOMAC Score (Western Ontario and McMaster Universities) 10

2. Health circuit in relation with knee osteoarthritis, from economic perspective 10

3. Quality of Life EQ-5D 11

4 - Methods and statistic parameters 12

A. Data collection and control 12

B. General analysis 12

C. Analysis of assessment criteria 12

5 - Results 13

A. Populations 13

B. WOMAC 14

C. Quality of Life 14

D. Inter group comparison 15

1. Evolution of hospitalizations 16

2. Evolution of X-ray examinations 17

3. Evolution of consultations 18

4. Evolution of drugs consumption 22

5. Evolution of life help devices 24

6. Evolution of stays in healthcare centers 25
7. Evolution of medical transportation 26
8. Evolution of sick leaves 26

6 - Conclusion 27

1 - OBJECTIVES AND STUDY DESIGN

A. Objectives of the study

Study title

Benefit and utility assessment in the management of knee osteoarthritis, in current practice, of intra-articular injections of a hyaluronic acid viscoelastic solution, ARTHRUM H 2%®

Scientific Committee

- Mme Marie-Paule SERRE (Professeur Université Paris VI)
- Professeur Thierry THOMAS (Service Rhumatologie CHU, Saint-Etienne)
- M. Pierre LEVY (Maître de conférences, Paris Dauphine)
- Docteur Françoise AMOUROUX (Pharmacien, Saint - Hélène)

Objectives of the study

Benefit – risk analysis
- To assess the consumption of non steroidal anti inflammatory drugs (NSAIDs)
- To estimate the iatrogenicity risk due to the consumption of non steroidal anti inflammatory drugs (NSAIDs)

Cost – utility analysis
- To measure the cost of all treatments, in current practice with non steroidal anti inflammatory drugs, and after or not therapeutic intervention with ARTHRUM H 2%®
- To assess the Quality of Life of the patient before or after therapeutic intervention with ARTHRUM H 2%® compared to the current practice with NSAIDs

Investigators

Dispensary pharmacists in metropolitan France

Main assessment criteria

The main assessment criteria for the study is to evaluate the percentage of patients under non steroidal anti inflammatory drugs during the follow-up period of the study. This criteria should allow to assess the impact of the therapeutic intervention with hyaluronic acid, on the consumption of non steroidal anti inflammatory drugs

B. Study design

Study in real current life, longitudinal and multicentric; comparative between knee osteoarthitis treatments by NSAIDs or after intervention of an intra articular viscoelastic solution ARTHRUM H 2%®

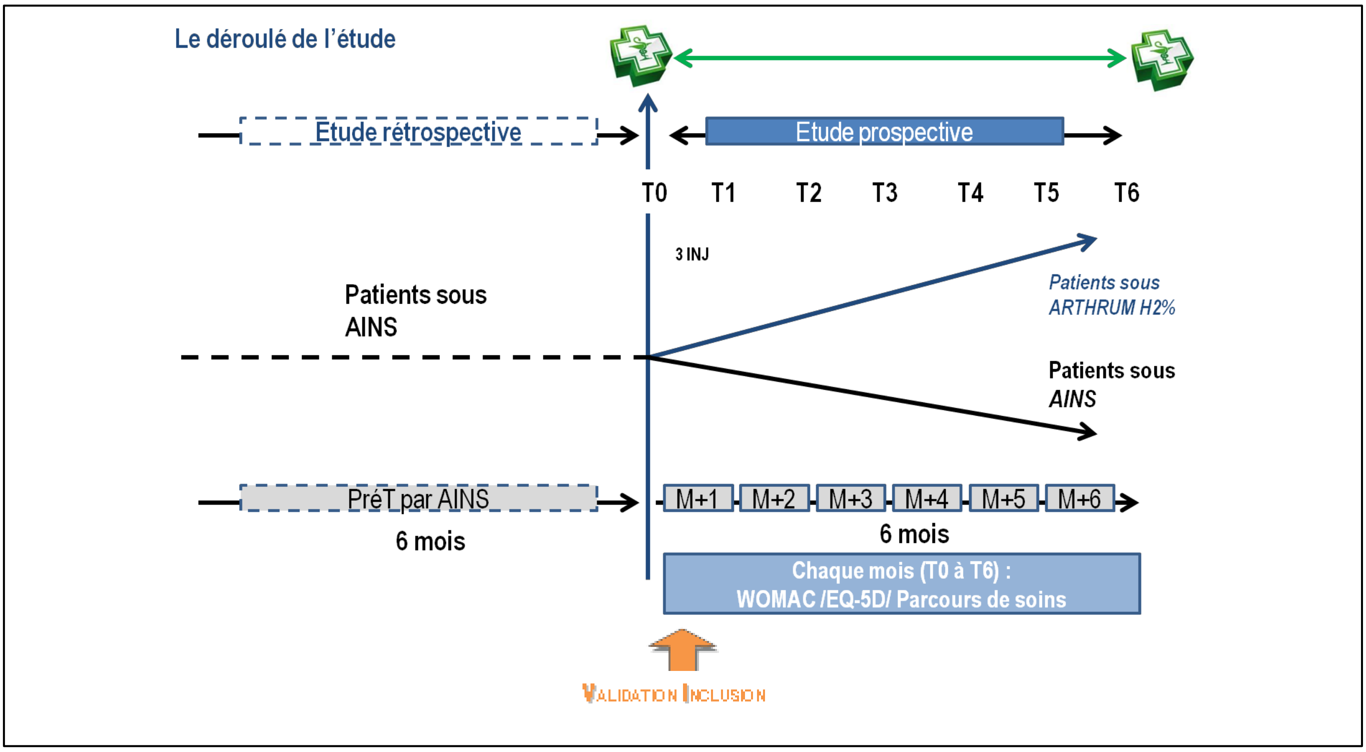

2 - Population and enrollment

For this study, 2 groups have been analyzed:

- Patients treated with NSAIDs, since more than 6 months, then ARTHRUM H 2%® at T0
- Patients treated with NSAIDs, since more than 6 months, and always treated with NSAIDs at T0

The patients responding to eligibility criteria and having given their consent by written, have been consecutively included by each pharmacist investigator. Each pharmacist has included at maximum, 1 patient ARTHRUM H 2%® and 1 patient under NSAIDs.

A. Recruitment of investigators

Pharmacists investigators have been recruited from a representative panel of 3,004 dispensary (town) pharmacies upon criteria of activity size and location, for the totality of pharmacies in metropolitan France (panel CELTIPHARM®).

A first sampling, stratified accordingly to the market of knee osteoarthritis, was performed to randomly select 700 pharmacies, among this panel.

The study has been proposed to all these defined pharmacists, and the recruitment has been stopped when the target number of pharmacists having accepted to participate as investigators, has been reached: 250 pharmacy centers in metropolitan France.

As for any study based on the contribution of voluntary investigators, a representativeness bias must be considered and estimated. For this, the profile of the pharmacist investigators having included at least one patient in the study, has been compared to the profile of the whole French pharmacists upon general criteria of size and location. It is also possible to compare this profile to the profile of all pharmacies having an activity for selling NSAIDs and/or ARTHRUM H 2%® treatments and belonging to the CELTIPHARM panel of 3,004 pharmacies in metropolitan France.

B. Enrollment of patients in real life

To validate the matching for patients included in the study, investigators had to respect the inclusion criteria.

The pharmacist investigators have orally informed the patients about medico-economic objectives of the study, and by written with acceptance signature on the informed consent form.

Only medico-economic objectives of the study have been described to the patients, to eliminate an obvious analysis bias from a potential Hawthorne effect

Following the signature of the informed consent form, the matching of populations has been obtained as following: the patient has filled, with the pharmacist assistance :

- **Patient profile** : age, sex of the patient, as well as his knee osteoarthritis grade, from his X-ray report,
- **Decision tree :** to validate eligibility criteria, for the patients included in the study,
- **WOMAC** Questionnaire, relative to the severity of his knee osteoarthritis: the WOMAC score is the most commonly used index to assess pain and functional handicap, in patients suffering from knee osteoarthritis.

This pre-inclusion step have allowed the pharmacists, to include eligible patients and to warrant the matching of populations between the observed groups.

Inclusion criteria

Inclusion criteria	Non-inclusion criteria
• Patient man or woman, aged from 40 to 75 years<br>• Patient with a KL grade 2 or 3 knee osteoarthritis<br>• Patient with a symptomatic knee osteoarthritis, justifying NSAIDs uptakes every month, since over 6 months<br>• Patient having an X-ray report, dated from less than 6 months, to confirm a knee osteoarthrosis<br>• Patient with a WOMAC score from 30 to 60, at inclusion (0-100 scale)<br>• Patient able to understand the requirements of the study and to give his consent by written, for his participation to the study<br>• Patient geographically stable during the whole duration of the study	• Patient with a bilateral knee osteoarthritis<br>• Patient with an infectious or non-infectious inflammatory knee arthritis<br>• Patient having anteriorly received a viscosupplementation treatment<br>• Patient unlikely to understand the conditions for assessment of the study criteria, or to be followed

200 patients ARTHRUM H2%® and 200 patients NSAIDs, for a total of 400 patients had to be included in the study.

This population for analysis has been made up from the patients enrolled by the pharmacists. Each having included at maximum one patient under NSAIDs and one patient treated with ARTHRUM H2%®, in the same investigator center.

No case of pharmacovigilance and/or materiovigilance has been reported.

3 - DATA COLLECTION

A. Population : Method for collecting data

Each pharmacist investigator has consecutively included patients meeting elegibility criteria for the study, and filled with them 7 paper data sheets and questionnaires totally anonymous (every 30 days)

Patients responding to eligibility criteria, and having given their oral consent, have been included by each pharmacist investigator. Moreover, each pharmacist investigator have completed, when needed, the non-inclusion register, to list main characteristics of the non-included patients, during the inclusion period of the study.

Patients have completed the data sheets and questionnaires, with assistance of the pharmacist investigator, just at the time of inclusion.
The data sheets and questionnaires have been sent by fax or email to a green number at the organization of the CELTIPHARM group.

B. Identification data for the population

Regarding included patients, their characteristics have been compared with the data contained in the non-inclusion register used for the study.
Moreover, the profile of the included patients, has been screened at the time of inclusion (T0, D0) for the following criteria:

- date of inclusion
- sex and age
- radiological antecedents: KL grade II or III
- assessment of the ongoing treatment at the time of inclusion and during the last 6 months
- WOMAC score
- decision tree to justify the inclusion regarding eligibility criteria

Comparisons have been done on main characteristics, sur as age, sex and treatment received.

C. Collected variables

Data have been collected at T0 (D0) / T1 (D30) / T2 (D60) / T3 (D90) / T4 (D120) / T5 (D150) / T6 (D180)

- T0 (D0) : Retrospective data from a 6-month history
- T1 (D30) / T2 (D60) / T3 (D90) / T4 (D120) / T5 (D150) / T6 (D180) : follow-up every month for study duration

To evaluate the effect of knee osteoarthritis in patient real life, and utility of a therapeutic intervention, it has been important to collect following information, through:

- An assessment method for health care and investment costs induced by the pathology
- An analytical assessment method including knee osteoarthritis characterization parameters: pain, stiffness and function
- An assessment method for consequences, evaluated at patient individual level

1. WOMAC score (Western Ontario and McMaster Universities)

WOMAC index is widely used in the evaluation of the severity of lower limbs osteoarthritis in humans. This index is a self-administrated questionnaire consisting of 24 items divided into 3 subscales:

- 5 questions for pain subscale
- 2 questions for stiffness subscale
- 17 questions for physical function

To quantify the WOMAC index, the patient has answered to each item, on a scale of 0 to 4, respectively:

- 0 None
- 1 Mild
- 2 Moderate
- 3 Severe
- 4 Extreme

Global score has been calculated by adding the 24 results. Then, it has been normalized on a base 100 scale, to obtain the WOMAC global score. Moreover each WOMAC subscale score has also been normalized on the 100 scale. It must be noted that higher the scores are, the more severe the knee osteoarthritis is.
Any missing data has been considered as a lack of answer, for an item which was not related to the patient. In this case, the missing data has been interpreted as a 0 score for the specific item.

2. Health care circuit, in relation with knee osteoarthritis, from an economical point of view

- Treatments delivered, in relation with knee osteoarthritis
- Medical and paramedical consultations, in relation with knee osteoarthritis
- Hospitalizations and interventions, in relation with knee osteoarthritis
- Sick leaves - Medical transportation, in relation with knee osteoarthritis

→**AMO (assurance maladie obligatoire):** national health insurance system

3. Quality of Life EQ-5D

The self-administrated questionnaire EQ-5D includes 5 questions each with 3 levels of answer:

- for mobility, self-care, usual activities, pain / discomfort, anxiety / depression

In addition to the questions, an evaluation of health state is proposed with a vertical analog scale (0 to 100).

Once all data collected, the global score has been calculated, then normalized on a 100 base, with the maximum score corresponding to the best possible Quality of Life for the patient.

4 - METHODS AND STATISTIC PARAMETERS

A. Data collection and control

The questionnaires have been transmitted by the pharmacists to a secured mail-box integrating a cryptage modulus, then data and questionnaires have been scanned. Data have been stored in a dedicated room, with control and access limited to only dedicated staff.
Keyboarding has been done by a team of operators, different from the team who performed the anonymization of the questionnaires:

- Keyboarding of the questionnaires has been done by the Department Direct Marketing of CELTIPHARM.
- A double capture has been done for 10% of the questionnaires for the Quality Assurance System of CELTIPHARM.

The data managers have proceed with the creation of the input masks, to the quality control and to the data freezing, in accordance with the procedures of CELTIPHARM.
A control of the captured data has been done to identify missing or incoherent data, at the time the questionnaires have been received.
Qualitative data have been recoded before analysis.

B. General analysis

Sampling method and statistic analysis have been performed with software R version 3.0.2.
Sub-groups have been analyzed. All the collected data have been described and analyzed.
The quantitative variables have been described by their mean, median, standard deviation, and extreme values (minimum and maximum).
The qualitative variables have been described by the frequency of each of their modalities.

C. Analysis of assessment criteria

Quantitative variables have been described by their mean, median, standard deviation, minimum and maximum. They have been described with the study of differential costs for drug and other expenses, with estimation of a ratio between the 2 studied groups.

The qualitative variables have been described by the frequency of each of their modalities.

The number of QALYs has been calculating by weighting from the time spent at each health state, using the associated scores to these states. They have been described with the study of QUALYs differences with estimation of a ratio between groups.

All variables have been submitted to a control of coherence and verified to optimize the quality of data from a statistic software previously validated.

The results obtained will be interpreted by the Scientific Committee, in order to identify the main criteria selected.

5 - RESULTS

A. Populations

Table 1 : Recruitment and population for analysis

Population		Recruited	Analyzed	
			N	%
**Investigators**	Pharmacists	252	238	94.44
**Patients included**	NSAIDs	213	199	93.42
	ARTHRUM H2%	216	202	93.51
	Total	*429*	*401*	*93.47*

28 patients have not been analyzed for following reasons:

- X-ray reports not transmitted N=7
- Non-respect of eligibility criteria N=5
- Lost to follow-up N=16

Table 2 : Characteristics of patients

Characteristics of patients		NSAIDs (N=199)	ARTHRUM H2% (N=202)
**Age (years)**	Mean±SD	62.3±8.2	65.6±7.8
	Min ; Max	40 ; 73	42 ; 75
**Sex**	Men	89 (45%)	83 (41%)
	Women	110 (55%)	119 (59%)
**KL radiological grade**	Grade II	103 (52%)	109 (54%)
	Grade III	96 (48%)	93 (46%)

B. WOMAC

WOMAC Scores	NSAIDs (N=199)			ARTHRUM H2% (N=202)			Difference T0 / T3		Difference T0 / T6	
	T0	T3	T6	T0	T3	T6	AINS (N=199)	ARTHRUM (N=202)	AINS (N=199)	ARTHRUM (N=202)
**WOMAC global,** Mean	49.2	47.6	43.6	49.7	35.4	28.7	1,6	14,3	5,6	21,0
SD	16.8	17.7	18.3	17.9	17.5	18.6	17.6	17.7	17.6	18.3
Min; Max	21;81	20;82	22;83	22;82	16;78	7;60	-	-	-	-
**WOMAC pain,** Mean	50.4	46.5	43.5	49.9	33.5	27.6	3.9	16.4	6.9	22.3
SD	16.1	17.3	18.1	17.2	17.9	18.2	16.7	17.6	17.1	17.7
Min; Max	24;78	21;76	23;78	26;75	17;71	8;68	-	-	-	-
**WOMAC stiffness,** Mean	45.8	43.3	41.1	45.7	31.9	26.2	2.5	13.8	4.7	19.5
SD	15.2	16;7	16.6	15.9	17.1	16.8	16.0	16.5	15.9	16.4
Min; Max	22;81	19;78	19;75	23;83	17;78	7;61	-	-	-	-
**WOMAC function, Mean**	47.5	45.1	41.8	48.1	38.5	29.3	2.4	9.6	5.7	18.8
SD	18.7	19.2	20.1	18.5	20.7	20.2	19.0	19.6	19.4	19.4
Min; Max	18;85	22;87	21;82	20;88	19;84	4;52	-	-	-	-

C. Quality of Life

**EQ-5D : Quality of Life**	**NSAIDs (N=199)**			**ARTHRUM H2% (N=202)**		
	T0	T3	T6	T0	T3	T6
**Health state**						
Mean ± SD	42±21	51 ±14	50 ±22	43 ±24	56 ±19	64 ±16
Min; Max	16 ; 94	12 ; 96	17 ; 91	14 ; 88	17 ; 91	23 ; 95

D. Inter group comparison

Inter-group comparison<br>ARTHRUM vs NSAIDs		Difference of index variation<br>(SD)<br>*base 0-100*	Effect size<br>[CI 95%]	p-value
WOMAC Global	T3	-12.7 (17.6)	-0.72 [-0.92 ; -0.52]	5.6E-13
	T6	-15.4 (17.9)	-0.86 [-1.06 ; - 0.66]	7.5E-18
WOMAC A (pain)	T3	-12.5 (17.1)	-0.73 [-0.93 ; - 0.53]	2.8E-13
	T6	-15.4 (17.4)	-0.88 [-1.08 ; - 0.68]	8.8E-19
WOMAC B (stiffness)	T3	-11. 3 (16.2)	-0.70 [-0.90 ; - 0.50]	3.3E-12
	T6	-14.8 (16.1)	-0.92 [-1.12 ; - 0.72]	4.2E-20
WOMAC C (function)	T3	-7.2 (19.3)	-0.37 [-0.57 ; - 0.17]	0.000001
	T6	-13.1 (19.4)	-0.68 [-0.88 ; - 0.48]	1.8E-22
Quality of Life<br>EQ-5D	T3	-4 (19.9)	-0.20 [-0.40 ; - 0.002]	0.044
	T6	-13 (21.0)	-0.62 [-0.82 ; - 0.42]	5.2E-10

1. Evolution of hospitalizations

Hospitalizations	NSAIDs (N=199)			ARTHRUM H2% (N=202)		
	T0	T3	T6	T0	T3	T6
Number of patients (%)<br>- ***Nb total admissions***<br>- ***Nb visits average per patient***	6 (3%)<br>8<br>0.04	2 (1%)<br>3<br>0.02	3 (1%)<br>3<br>0.02	7 (3%)<br>7<br>0.04	2 (2%)<br>3<br>0.01	0 (0%)<br>0<br>0.00
Cost of hospitalizations	4 720.00 €	1 770.00 €	770.00 €	4 130.00 €	1 770.00 €	0.00 €
Average cost per patient	23.71 €	8.90 €	8.90 €	20.44 €	8.76 €	0.00 €

The costs correspond to the part covered by "Assurance Maladie Obligatoire" of the national health insurance system

We observe a lower rate of admissions with ARTHRUM H 2%, but the difference between groups is not significant (p =0.44)

2. Evolution of X-ray examinations

X-ray examinations	NSAIDs (N=199)			ARTHRUM H 2% (N=202)		
	T0	*T3*	*T6*	*T0*	*T3*	*T6*
- Number of patients (%)	*199(100%)*	*6(3%)*	*3(14%)*	*202(100%)*	*2(1%)*	*2(9.4%)*
- Total number of X-ray exams (%)	*223(112%)*	*6(3%)*	*3(16%)*	*215(106%)*	*3(1%)*	*2(10.9%)*
- Average number per patient	*1.12*	*1.00*	*1.00*	*1.06*	*1.50*	*1.00*
Total cost of X-ray exams	*6021.00 €*	*135.00 €*	*81.00 €*	*5805.00 €*	*81.00 €*	*54.00 €*
Average cost per patient	*30.25 €*	*0.81 €*	*0.40 €*	*29.17 €*	*0.40 €*	*0.27 €*

The costs correspond to the part covered by "Assurance Maladie Obligatoire"

We do not observe a significant difference between groups, for X-ray exams (p>0.668).

3. Evolution of consultations

Medical consultations	NSAIDs (N=199)			ARTHRUM H 2% (N=202)		
General practitioners (GP)	T0	T3	T6	T0	T3	T6
- Number of patients (%)<br>*- Total number of visits*<br>*- Average number per patient*	23 (62%)<br>296<br>1.48	22 (11%)<br>31<br>0.16	35 (18%)<br>42<br>0.21	45 (72%)<br>309<br>1.53	5 (3%)<br>6<br>0.03	9 (5%)<br>12<br>0.06
Cost of consultations to GP	6808 €	713 €	966 €	7107 €	138 €	276 €
Average cost per patient	34.21 €	3.58 €	4.85 €	35.18 €	0.68 €	1.37 €

The costs correspond to the part covered by "Assurance Maladie Obligatoire"

We observe a significant reduction of the consultations to general practitioners in the group ARTHRUM H 2% (p = 7.068 E-7).

Medical consultations	NSAIDs (N=199)			ARTHRUM H 2% (N=202)		
Rheumatologists	T0	T3	T6	T0	T3	T6
- Number of patients (%)	196 (98%)	56 (28%)	65 (33%)	202 (100%)	202 (100%)	93 (46%)
- Total number of visits	264	73	82	272	348	103
- Average number per patient	1.32	0.36	0.41	1.34	1.72	0.51
Cost of consultations to rheumatologists	7392 €	2044 €	2296 €	7616 €	9744 €	2884 €
Average cost per patient	37.15 €	10.27 €	11.53 €	37.70 €	48.24 €	14.27 €

The costs correspond to the part covered by "Assurance Maladie Obligatoire"

We observe a significant increase of the consultations to rheumatologist in the group ARTHRUM H 2% (p=2.48 E-4).

AFCDP
.TIPHARM est membre de la Fédération Nationale des Tiers de Confiance
fntc

Medical consultations	NSAIDs (N=199)			ARTHRUM H 2% (N=202)		
Other specialists	T0	T3	T6	T0	T3	T6
- Number of patients (%)<br>*- Total number of visits*<br>*- Average number per patient*	38 (19%)<br>39<br>0.20	4 (2%)<br>5<br>0.03	14 (7%)<br>16<br>0.08	41 (20%)<br>46<br>0.22	15 (7%)<br>24<br>0.12	6 (3%)<br>9<br>0.04
Cost of consultations to other specialists	1326 €	170 €	544 €	1564 €	816 €	306 €
Average cost per patient	6.66 €	0.85 €	2.73 €	7.74 €	4.04 €	1.51 €
Total cost per patient Medical consultations	78.02 €	14.70 €	19.11 €	80.62 €	52.96 €	17.15 €

The costs correspond to the part covered by "Assurance Maladie Obligatoire"

We do not observe a significant difference between groups for medical consultations to other specialists. (p=0.1).

Paramedical consultations	NSAIDs (N=199)			ARTHRUM H 2% (N=202)		
Physiotherapists / Osteopaths	T0	T3	T6	T0	T3	T6
- Number of patients (%)	19 (10%)	14 (7%)	16 (8%)	17 (8%)	6 (3%)	9 (5%)
- Total number of visits	24	16	19	19	7	12
- Average number per patient	0.12	0.08	0.10	0.09	0.03	0.06
Cost of paramedical consultations	384 €	256 €	304 €	304 €	112 €	192 €
Average cost per patient	1.92 €	1.29 €	1.53 €	1.50 €	0.55 €	0.95 €

The costs correspond to the part covered by "Assurance Maladie Obligatoire"

We do not observe a significant difference between groups for paramedical consultations (p= 0.721).

4. Evolution of drugs consumption

Drug treatments	NSAIDs (N=199)			ARTHRUM H 2% (N=202)		
ARTHRUM H 2%	T0	T3	T6	T0	T3	T6
Number of patients (%)<br>*Average number of units per patient*	0 (0%)<br>0	0 (0%)<br>0	0 (0%)<br>0	0 (0%)<br>0	202 (100%)<br>1	0 (0%)<br>0
Average cost per patient	0.00 €	0.00 €	0 €	0 €	30 €*	0 €
NSAIDs	T0	T3	T6	T0	T3	T6
Number of patients (%)<br>*Average number of units per patient*	199 (100%)<br>14.4	199 (100%)<br>7.3	199 (100%)<br>7.9	202 (100%)<br>12.1	133 (67%)<br>6.4	89 (44%)<br>6.7
Average cost per patient	45.36 €	20.84 €	22.48 €	38.18 €	11.96 €	8.39 €
Analgesics	T0	T3	T6	T0	T3	T6
Number of patients (%)<br>*Average number of units per patient*	90 (45%)<br>7.2	94 (47%)<br>3.3	90 (45%)<br>3.5	145 (72%)<br>11.4	123 (61%)<br>3.9	105 (52%)<br>3.1
Average cost per patient	9.92 €	4.53 €	4.94 €	15.71 €	5.37 €	4.26 €
Corticoids	T0	T3	T6	T0	T3	T6
Number of patients (%)<br>*Average number of units per patient*	42 (21%)<br>6.6	56 (28%)<br>3.2	58 (29%)<br>3.0	46 (23%)<br>6.3	38 (19%)<br>3.0	18 (9%)<br>3.1
Average cost per patient	6.26 €	3.11 €	3.07 €	6.18 €	3.05 €	3.09 €

Drug treatments	NSAIDs (N=199)			ARTHRUM H 2% (N=202)		
Proton pump inhibitors	T0	T3	T6	T0	T3	T6
Number of patients (%)<br>*Average number of units per patient*	50 (25%)<br>13.4	62 (31%)<br>6.6	58 (29%)<br>6.9	42 (21%)<br>6.0	30 (15%)<br>3.3	12 (6%)<br>3.0
Average cost per patient	6.9 €	5.6 €	6.1 €	5.1 €	3.9 €	2.9 €
SYSADOA	T0	T3	T6	T0	T3	T6
Number of patients (%)<br>*Average number of units per patient*	74 (37%)<br>6.7	72 (36%)<br>3.2	72 (36%)<br>3.2	79 (39%)<br>6.9	69 (34%)<br>3.1	69 (30%)<br>3.0
Average cost per patient	27.9 €	13.6 €	13.9 €	28.6 €	14.9 €	13.7 €

Total :

Drug treatments (total)	NSAIDs (N=199)			ARTHRUM H 2% (N=202)		
Number of patients (%)<br>*Average number of units per patient and per month*	199 (100%)<br>3.1±1.3	199 (100%)<br>2.6±1.9	199 (100%)<br>3.5±1.1	202 (100%)<br>2.9±1.7	202 (100%)<br>4.2±1.2	160 (79%)<br>1.9±1.3
*Average cost per patient **	96.34 €	47.68 €	50.49 €	93.77 €	69.18 €	32.34 €

The costs correspond to the part covered by "Assurance Maladie Obligatoire"

(*) For ARTHRUM H 2% : 50% of the annual cost (60€) are considered for this 6-month study.
ARTHRUM H 2% Treatment was considered at early T1, to allow comparison at T0 between the groups

At T0, we do not observe any significant difference between groups.

At T3, we observe a significant difference. only linked to the impact of ARTHRUM H 2% treatment.

At T6, drug consumption is significantly reduced for the group ARTHRUM H 2% (p=0.002).

For the 6-month period (T1-T6), no difference is observed for the total cost per group, meaning that the cost of ARTHRUM H 2% is compensated by savings on other drugs.

5. Evolution of life help devices

Life help devices	NSAIDs (N=199)			ARTHRUM H 2% (N=202)		
	T0	T3	T6	T0	T3	T6
Number of patients (%)	*18 (9%)*	*10 (5%)*	*12 (6%)*	*15 (7%)*	*7 (3%)*	*5 (2%)*
- Insoles and shoes	*3*	*0*	*0*	*2*	*1*	*1*
- Sticks	*10*	*9*	*10*	*9*	*6*	*3*
- Wheelchairs	*2*	*0*	*0*	*1*	*0*	*0*
- Knee orthesis	*2*	*1*	*1*	*3*	*1*	*1*
- Walkers	*1*	*0*	*1*	*0*	*0*	*0*
Cost of life help devices	*1073.00 €*	*129.00 €*	*171.00 €*	*656.00 €*	*111.00 €*	*93.00 €*
Average cost per patient	*5.39 €*	*0.65 €*	*0.86 €*	*3.25 €*	*0.55 €*	*0.46 €*

The costs correspond to the part covered by "Assurance Maladie Obligatoire"

We do not observe a significant difference between groups for life help devices (p=0.98).

6. Evolution of stays in healthcare centers

Stays in healthcare centers	NSAIDs (N=199)			ARTHRUM H 2% (N=202)		
	T0	T3	T6	T0	T3	T6
Number of patients (%)	**1 (0.5%)**	**2 (1%)**	**1 (0.5%)**	**2 (1%)**	**0 (0%)**	**1 (0.5%)**
Thermal cure	***1***	***2***	***1***	***2***	***0***	***1***
Rest home	***0***	***0***	***0***	***0***	***0***	***0***
Retirement home	***0***	***0***	***0***	***0***	***0***	***0***
Other						
Cost of stays in healthcare centers	**510.51 €**	**1 021.00 €**	**510.51 €**	**510.51 €**	**0.00 €**	**510.51 €**
Average cost per patient	**2.57 €**	**5.14 €**	**2.57 €**	**2.57 €**	**0.00 €**	**2.57 €**

The costs correspond to the part covered by "Assurance Maladie Obligatoire"

We do not observe a significant difference between groups for stays in healthcare centers (p= 0.41).

7. Evolution of medical transportation

Medical transportation	NSAIDs (N=199)			ARTHRUM H 2% (N=202)		
	T0	T3	T6	T0	T3	T6
Number of patients	3	2	2	4	2	0
Average number of transportation	*12,5*	*1,5*	*1,5*	*10*	*2*	*0*
Cost of transportation	61.70 €	49.75 €	49.75 €	72.70 €	56.60 €	0.00 €
Average cost per patient	0.31 €	0.25 €	0.25 €	0.36 €	0.28 €	0.00 €

The costs correspond to the part covered by "Assurance Maladie Obligatoire"

We do not observe a significant difference between groups for medical transportation (p= 0.736).

8. Evolution of sick leaves

Sick leaves	NSAIDs (N=199)			ARTHRUM H 2% (N=202)		
	T0	T3	T6	T0	T3	T6
Number of patients	20	19	21	18	11	8
Active patients %	*21%*	*21%*	*21%*	*28%*	*28%*	*28%*
Total days sick leaves (days)	*378*	*186*	*198*	*356*	*153*	*107*
Average duration (days)	*16*	*18*	*17*	*17*	*14*	*11*
Cost of sick leaves	13 715 €	5 562 €	5 821 €	13 025 €	5 175 €	3 579 €
Average cost per patient	68.91€	27.95 €	29.25 €	64.48 €	25.62 €	17.72 €

The costs correspond to the part covered by "Assurance Maladie Obligatoire"

We observe a significant reduction of sick leaves in the group ARTHRUM H 2%, past the first 3-month following the treatment (p=0.0062).

6 - CONCLUSION

Main criteria – Benefit risk analysis

We observe a reduction of the number of patients under NSAIDs in the group ARTHRUM H 2%, and also a reduction of NSAIDs consumption per patient in same group.

Drug treatments	NSAIDs (N=199)			ARTHRUM H 2% (N=202)		
NSAIDs	T0	T3	T6	T0	T3	T6
Number of patients (%)<br>*Average number of units per patient*	199 (100%)<br>14.4	199 (100%)<br>7.3	199 (100%)<br>7.9	202 (100%)<br>12.1	133 (67%)<br>6.4	89 (44%)<br>6.7
Average cost per patient	**45.36 €**	**20.84 €**	**22.48 €**	**38.18 €**	**11.96 €**	**8.39 €**

Assessment of expenses of the healthcare circuit

After T0, we observe variations for several expenditure items, between the groups NSAIDs and ARTHRUM H 2%.

Average cost per patient	NSAIDs (N=199)			ARTHRUM H 2% (N=202)		
	T0	T3	T6	T0	T3	T6
Hospitalizations	23.71 €	8.90 €	8.90 €	20.44 €	8.76 €	0.00 €
X-ray examinations	30.25 €	0.81 €	0.40 €	29.17 €	0.40 €	0.27 €
General practitioners consultations	34.21 €	3.58 €	4.85 €	35.18 €	0.68 €	1.37 €
Rheumatologists consultations	37.15 €	10.27 €	11.53 €	37.70 €	48.24 €	14.27 €
Other MD consultations	6.66 €	0.85 €	2.73 €	7.74 €	4.04 €	1.51 €
Paramedical consultations	1.92 €	1.29 €	1.53 €	1.50 €	0.55 €	0.95 €
Drug treatments	96.34 €	47.68 €	50.49 €	93.77 €	69.18 €	32.34 €
Life help devices	5.39 €	0.65 €	0.86 €	3.25 €	0.55 €	0.46 €
Stays in healthcare centers	2.57 €	5.14 €	2.57 €	2.57 €	0.00 €	2.57 €
Medical transportation	0.31 €	0.25 €	0.25 €	0.36 €	0.28 €	0.00 €
Sick leaves	68.91€	27.95 €	29.25 €	64.48 €	25.62 €	17.72 €
Average global cost per patient	307.42 €	107.37 €	113.36 €	296.16 €	158.30 €	71.46 €

Cost utility analysis

Before T0, there is no significant difference between the two groups, which are following identical treatments in parallel.
Then at T3, we observe a difference of 51 € between the 2 groups, whose an important part is linked to the difference of treatment costs, with incidence of ARTHRUM H 2% injections.
Finally at T6, we observe a significant reduction of the costs for the ARTHRUM H 2% group, in such a way that the global expenses remains similar between the two groups : **528 €** for NSAIDs vs **526 €** for ARTHRUM H 2%.

The impact of the sodium hyaluronate therapeutic intervention (ARTHRUM H 2% - 3 injections / year) allows therefore to reduce the consumption of non-steroidal anti anti-inflammatory drugs (NSAIDs), without inducing any over-cost for the national health insurance system ("assurance maladie obligatoire" = AMO).

Global cost assessment	NSAIDs (N=199)			ARTHRUM H 2% (N=202)		
	T0	T3	T6	T0	T3	T6
Average global cost per patient	307.42 €	107.37 €	113.36 €	296.16 €	158.30 €	71.46 €

528.15 € (NSAIDs total) **525.92 €** (ARTHRUM H 2% total)

Secondary objective - Symptoms and Quality of Life

With ARTHRUM H 2% (3 injections per year) treatment, we observe a significant and clinically important improvement on the severity of the symptoms (pain, function), with a clear improvement of the patients Quality of Life as direct consequence.

Results at T6 for symptoms and Quality of Life	Score variations since T0		Intergroup comparison ARTHRUM H 2% vs AINS (negative results are to the advantage of injected hyaluronic acid)			
	NSAIDs (N=199)	ARTHRUM H 2% (N=202)	Difference	Effect size ES	Effect size CI 95%	p-value
Pain, stiffness, function						
WOMAC A	-6.9	-22.3	-15.4	-0.88	[-1.08 ; -0.68]	< 0.000001
WOMAC B	-4.7	-19.5	-14.8	-0.92	[-1.12 ; -0.72]	< 0.000001
WOMAC C	-5.7	-18.8	-13.1	-0.68	[-0.88 ; -0.48]	< 0.000001
WOMAC global	-5.6	-21.0	-15.4	-0.86	[-1.06 ; -0.66]	< 0.000001
Quality of Life						
EQ-5D	8	21	-13	-0.62	[-0.82 ; -0.42]	< 0.000001
